# Supplementary material for: Nuclear and mitochondrial DNA editing in human cells with zinc finger deaminases
Source: Nat Commun. 2022 Jan 18;13:366. doi: 10.1038/s41467-022-27962-0 (PMC8766470; doi:10.1038/s41467-022-27962-0)
Supplement: Supplementary file 1 — Supplementary Information [file 41467_2022_27962_MOESM1_ESM.pdf]

Supplementary Information

Nuclear and mitochondrial DNA editing in human cells using zinc finger deaminases

Table of Contents

Supplementary Figures

Supplementary figure 1. Optimization of ZFD base editing using pTarget plasmids.
Supplementary figure 2. Effects of ZFD linker length on the base editing efficiency.
Supplementary figure 3. Designing the ZFD pairs by modifying previously-characterized ZFNs.
Supplementary figure 4. Possible ZFD architectures.
Supplementary figure 5. Indel frequencies generated by ZFDs targeted to endogenous sites.
Supplementary figure 6. ZFD protein purification and in vitro activity.
Supplementary figure 7. Possible mitoZFD architectures.
Supplementary figure 8. Editing in single cell-derived clones from populations of mitoZFD-treated HEK 293T cells.
Supplementary figure 9. Analysis of allele frequencies in single cell-derived clones from a population of ND1-mitoZFD-treated HEK 293T cells.
Supplementary figure 10. Analysis of allele frequencies in single cell-derived clones from a population of ND2-mitoZFD-treated HEK 293T cells.
Supplementary figure 11. Base editing activities of mitoZFDs, TALE-based DdCBEs, and ZFD/DdCBE hybrid pairs for the *COX2* site.
Supplementary figure 12. Mitochondrial genome-wide target specificity of the ND1-targeted mitoZFD depends on the concentration of the ZFD-encoding mRNA or plasmid.
Supplementary figure 13. Analysis of on- and off-target activity of *ND1*-targeted mitoZFD.
Supplementary figure 14. Mitochondrial genome-wide target specificity of the *ND2*-targeted mitoZFD depends on the concentration of the ZFD-encoding mRNA or plasmid.
Supplementary figure 15. Analysis of the on- and off-target activity of *ND2*-targeted mitoZFD.
Supplementary figure 16. Sequence logos with off-target sites of *ND2*-targeted mitoZFD.
Supplementary figure 17. Potential off-target sites of mitoZFD in nuclear DNA.

Supplementary Tables

Supplementary table 1. Spacer sequences, ranging from 1-24 bps in length, in the pTarget plasmids.

Supplementary table 2. Amino acid sequences of ZFD constructs used for the optimization of the ZFD architecture.

Supplementary table 3. Amino acid sequences of ZFD constructs for endogenous target sites.

Supplementary table 4. DNA sequences of zinc finger binding sites and spacer region of ZFDs for endogenous target sites.

Supplementary table 5. Amino acid sequences of mitoZFDs.

Supplementary table 6. DNA sequences of zinc finger binding sites and the spacer region of mitoZFDs.

Supplementary table 7. ZFD-GST sequences for protein purification.

Supplementary table 8. The DNA sequence of the PCR amplicon for the in vitro activity test of the ZFD protein.

Supplementary table 9. Amino acid sequence of *COX2* targeting DdCBE.

Supplementary table 10. List of primers used for targeted deep sequencing.

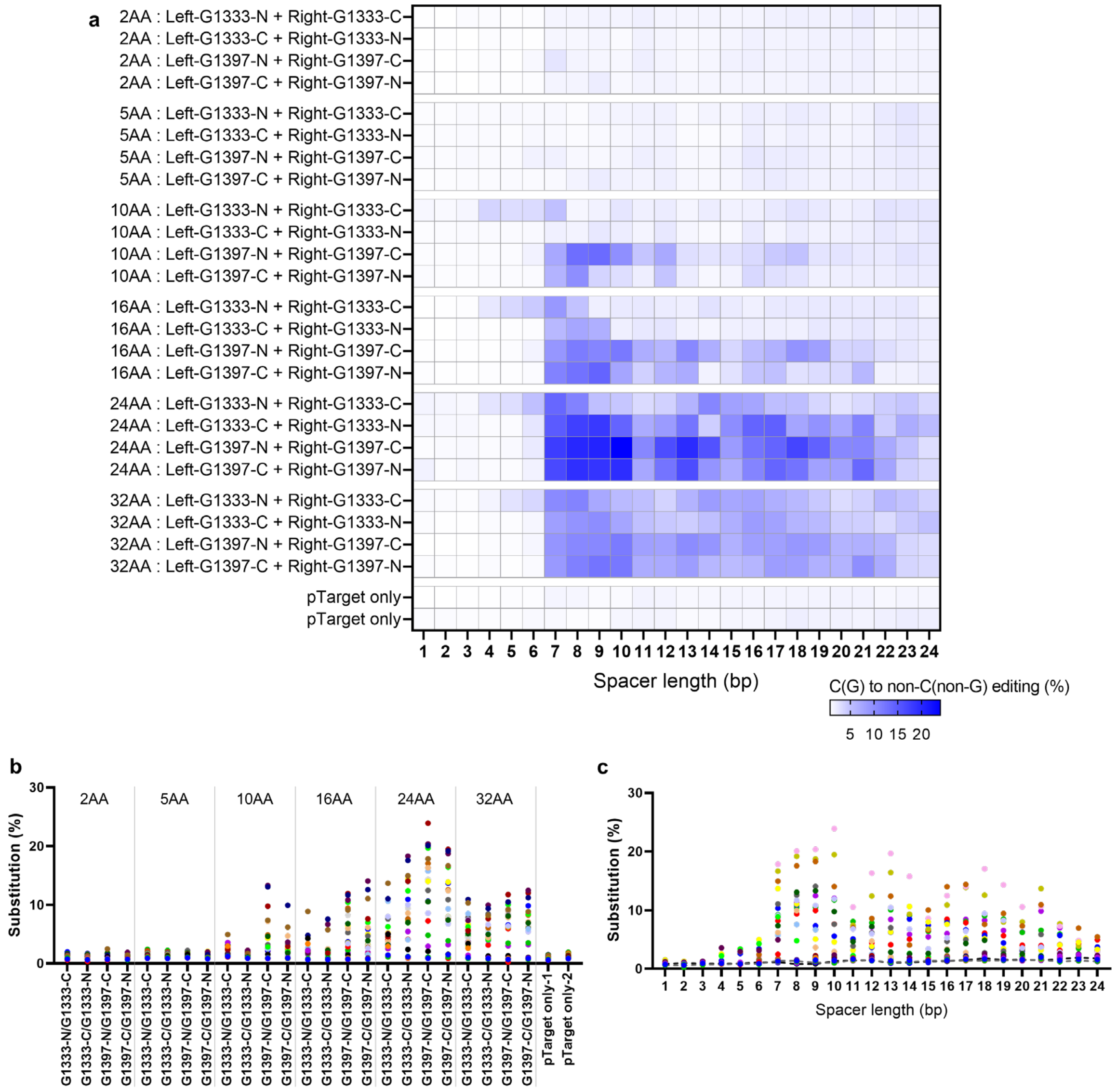

Supplementary figure 1. Optimization of ZFD base editing using pTarget plasmids. **a,** The frequency of C/G-to-non-C/G edits in pTarget spacers, which varied in length from 1-24 bps, depicted in a heat map. A variety of ZFD constructs were tested, which varied in the number of amino acids in the linker between the ZFP and the split $DddA_{tox}$ half and the site at which $DddA_{tox}$ was split. **b,** The overall activity of each ZFD pair is shown. In the nomenclature used for the x-axis labels, the left finger is listed first and the right finger is listed second. **c,** The frequency of C/G-to-non-C/G edits as a function of the spacer length. "AA" refers to the number of amino acids in the linker. Data are shown as means from n = 2 biologically independent samples. Source data are provided as a Source Data file.

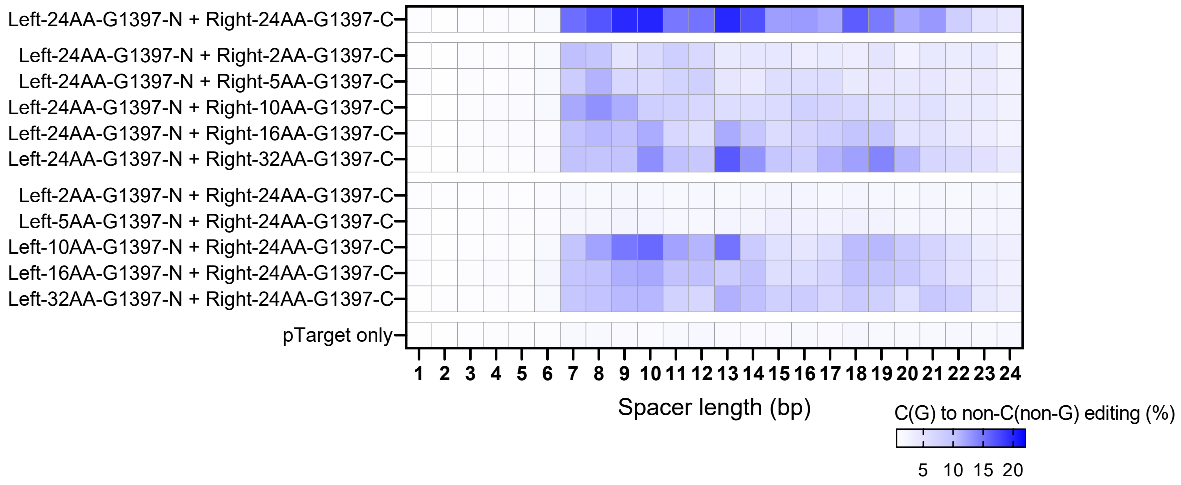

Supplementary figure 2. Effects of ZFD linker length on the base editing efficiency. Effect of the ZFD linker length on the frequency of C/G-to-non-C/G edits depicted in a heat map. The left member of the ZFD pair contained a fixed with the 24 AA linker and the right member contained linkers of variable length, or vice versa. Data are shown as means from n = 2 biologically independent samples. Source data are provided as a Source Data file.

CCR5-1 (ZFN)

```
                 1 2 3 4 5      4xZF
5'-GTCATCCTCATC  CTGAT  AAACTGCAAAAGGCT-3'
3'-CAGTAGGAGTAG  GACTA  TTTGACGTTTTCCGA-5'
       4xZF
```

CCR5-2 (ZFD)

```
                 1 2 3 4 5 6 7 8      4xZF
5'-GTCATCCTCATC  CTGATAAA  CTGCAAAAGGCT-3'
3'-CAGTAGGAGTAG  GACTATTT  GACGTTTTCCGA-5'
       4xZF
```

TRAC-CC (ZFN)

```
                        1 2 3 4 5      5xZF
5'-GTGATTGGGTTCCGAATC  CTCCT  CCTGAAAGTGGCCGG-3'
3'-CACTAACCCAAGGCTTAG  GAGGA  GGACTTTCACCGGCC-5'
         6xZF
```

TRAC-CC (ZFD)

```
                 1 2 3 4 5 6 7 8 9 10 11      5xZF
5'-GTGATTGGGTTC  CGAATCCTCCT  CCTGAAAGTGGCCGG-3'
3'-CACTAACCCAAG  GCTTAGGAGGA  GGACTTTCACCGGCC-5'
       4xZF
```

TRAC-NC (ZFN)

```
   6xZF + 1bp-skipping  1 2 3 4 5 6      6xZF
5'-TGTCAGTGATTGGGTTCCG  AATCCT  CCTCCTGAAAGTGGCCGG-3'
3'-ACAGTCACTAACCCAAGGC  TTAGGA  GGAGGACTTTCACCGGCC-5'
```

TRAC-NC (ZFD)

```
   5xZF + 1bp-skipping  1 2 3 4 5 6 7 8 9 10 11 12      5xZF
5'-TGTCAGTGATTGGGTT  CCGAATCCTCCT  CCTGAAAGTGGCCGG-3'
3'-ACAGTCACTAACCCAA  GGCTTAGGAGGA  GGACTTTCACCGGCC-5'
```

Supplementary figure 3. Designing the ZFD pairs by modifying previously-characterized ZFNs.

To make ZFD pairs with spacer regions of at least 7bps, previously-characterized ZFNs pairs were modified by deleting one or two zinc fingers or adding a few zinc fingers. ZFP-binding sites are underlined and shown in green.

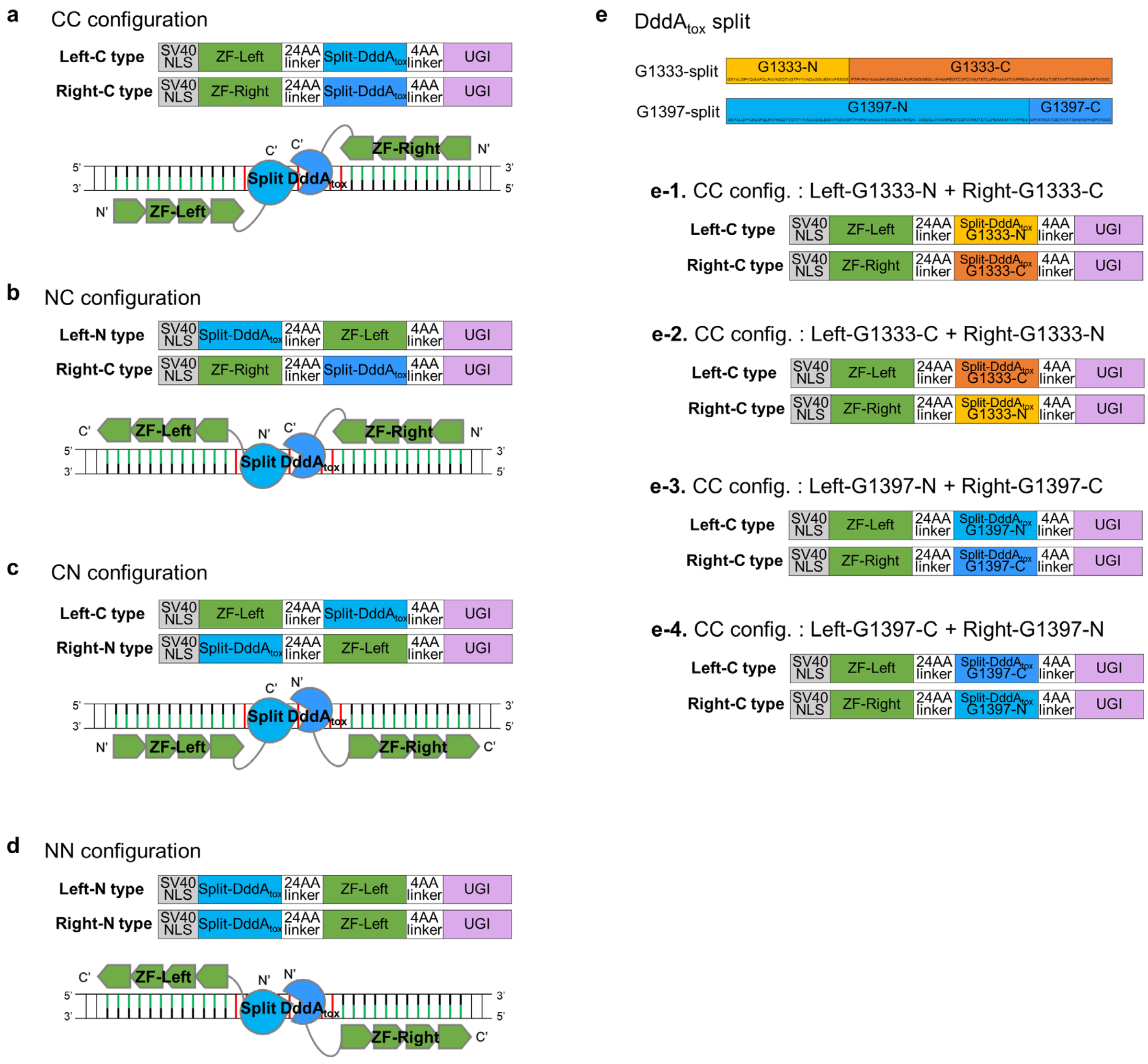

Supplementary figure 4. Possible ZFD architectures and DddA$_{tox}$ orientations.
a-d, Four potential ZFD configurations. The NC and CN configurations are structurally identical, but the types of left and right ZFD constructs are different. **e,** Four ZFD constructs, in which different DddA$_{tox}$ split orientations are used with the left and right zinc fingers.

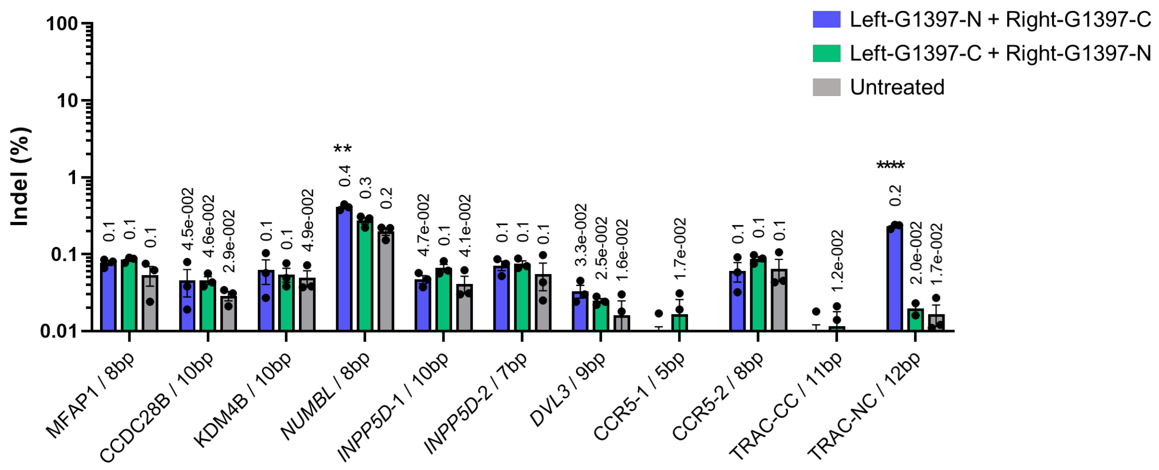

Supplementary figure 5. Indel frequencies generated by ZFDs targeted to endogenous sites.

All tested ZFDs generated indels at a frequency < 0.4%. All statistical analysis for comparing with untreated samples was conducted using unpaired Student's t-test (two-tailed) in GraphPad Prism 8. Statistical significance as compared with untreated samples was denoted with * = $p \leq 0.05$, ** = $p \leq 0.01$, *** = $p \leq 0.001$, **** = $p \leq 0.0001$, n.s. (not significant) = $p > 0.05$. p values of Left-G1397-N + Right-G1397-C form of *NUMBL* and *TRAC*-NC are 0.00226 and 0.00003. Data are shown as means with standard error of the mean (s.e.m.) from n=3 biologically independent samples. Source data are provided as a Source Data file.

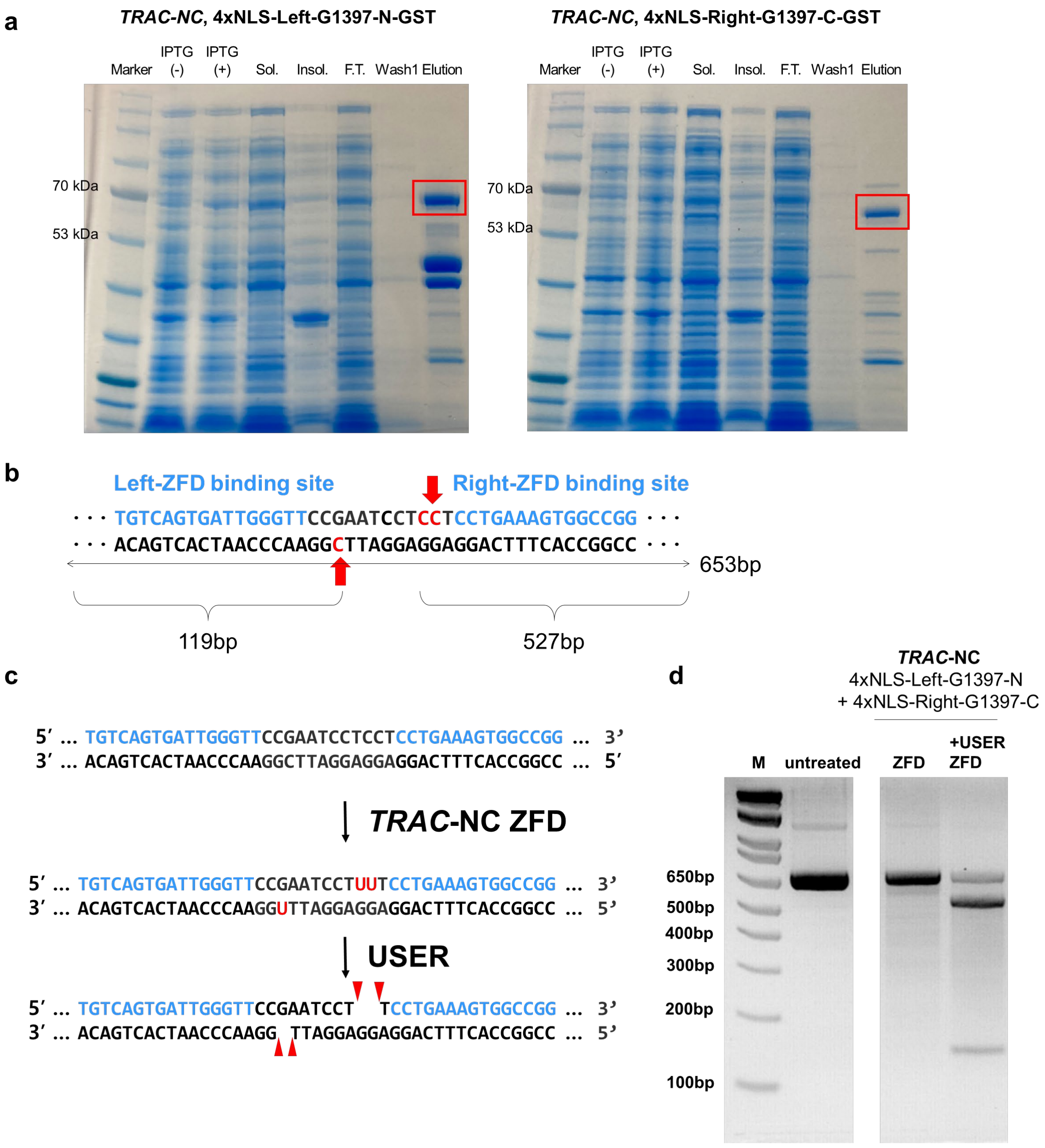

Supplementary figure 6. ZFD protein purification and in vitro activity.
a, Purification steps for a ZFD pair targeted to the *TRAC* site. GST-tagged proteins were purified from *E. coli* cell lysates using glutathione sepharose beads; purification steps were monitored using polyacrylamide gel electrophoresis. The gels were stained with Coomassie blue. Lane 1, molecular weight markers. Lane 2, Sample from cells in which protein expression was not induced with IPTG. Lane 3, Sample from cells in which protein expression was induced with IPTG. Lane 4, soluble fraction after sonication. Lane 5, insoluble fraction after sonication. Lane 6, flow-through fraction from the column. Lane 6, wash fraction. Lane 7, elution fraction. The sizes of representative markers are indicated on the left. The red box indicates the ZFD protein. Similar results were obtained in three replicates during the protein purification process. **b,** Left and right ZFD binding sites. The red arrows indicate possible sites for ZFD-induced deamination. **c,** Overview of ZFD activity on the PCR amplicon containing the *TRAC* site. The *TRAC*-NC ZFD pair first deaminates cytosines, generating uracils (indicated in red). The USER enzyme then excises the uracils, generating gaps (indicated by

the red triangles). **d,** Untreated PCR amplicons (left) and PCR amplicons treated with the ZFD pair (right) analyzed by agarose gel electrophoresis. The uncropped version of the gel image is provided in the Source Data file. Similar results were obtained in two replicates.

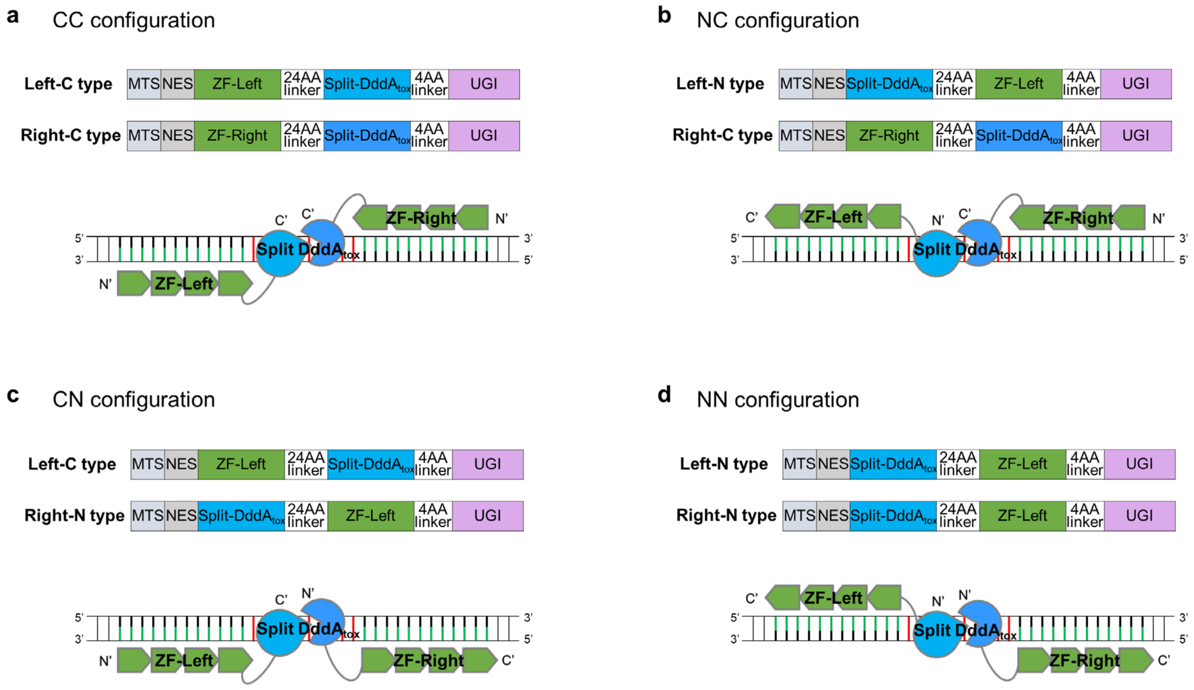

Supplementary figure 7. Possible mitoZFD architectures.
a-d, Four potential mitoZFD configurations. The NLS in conventional ZFDs has been replaced with an MTS and NES. The NC and CN configurations are structurally identical, but the types of left and right ZFD constructs are different.

a

MT-ND1
GTTTACTCAATC CTCTGATC$_8$ AGGGTGAGCATC
CAAATGAGTTAG GAGAC$_5$TAG TCCCACTCGTAG

	sub (%)		sub (%)
ND1-1	0.6	ND1-16	83.2
ND1-2	0.4	ND1-17	0.4
ND1-3	34.8	ND1-18	0.5
ND1-4	0.6	ND1-19	0.4
ND1-5	0.5	ND1-20	0.5
ND1-6	0.6	ND1-21	0.5
ND1-7	0.5	ND1-22	0.6
ND1-8	0.5	ND1-23	0.5
ND1-9	0.5	ND1-24	0.7
ND1-10	98.1	ND1-25	0.5
ND1-11	0.5	ND1-26	0.5
ND1-12	0.5	ND1-27	47.7
ND1-13	0.5	ND1-28	0.6
ND1-14	0.6	ND1-29	0.6
ND1-15	0.6	ND1-30	94.7

b

MT-ND2
CTACGCCTAATC TACTC$_5$C$_6$AC$_8$C$_9$TCAA TCACACTACTCC
GATGCGGATTAG ATGAGGTGGAGTT AGTGTGATGAGG

	sub (%)		sub (%)
ND2-1	0.6	ND2-19	0.9
ND2-2	0.7	ND2-20	0.7
ND2-3	0.7	ND2-21	67.5
ND2-4	0.6	ND2-22	1.0
ND2-5	0.6	ND2-23	71.3
ND2-6	0.7	ND2-24	75.7
ND2-7	0.6	ND2-25	0.6
ND2-8	0.6	ND2-26	0.7
ND2-9	0.7	ND2-27	0.7
ND2-10	0.9	ND2-28	0.8
ND2-11	0.7	ND2-29	0.7
ND2-12	0.8	ND2-30	0.6
ND2-13	0.6	ND2-31	45.1
ND2-14	0.7	ND2-32	57.9
ND2-15	0.6	ND2-33	52.6
ND2-16	0.7	ND2-34	25.5
ND2-17	0.7	ND2-35	0.9
ND2-18	0.6	ND2-36	0.8

c

	ND1 region sub (%)	ND2 region sub (%)
untreated-1	0.5	0.8
untreated-2	0.6	0.7
untreated-3	0.6	0.7
untreated-4	1.1	0.8
untreated-5	0.5	0.7
untreated-6	0.5	0.7
untreated-7	0.6	0.9
untreated-8	0.5	0.8
untreated-9	0.5	0.9
untreated-10	0.6	0.8
untreated-11	0.5	0.9
untreated-12	0.6	0.7
untreated-13	0.5	0.7
untreated-14	0.6	0.7
untreated-15	0.5	0.7
untreated-16	0.5	0.8
untreated-17	0.5	0.9
untreated-18	0.4	0.6

Supplementary figure 8. Editing in single cell-derived clones from populations of mitoZFD-treated HEK 293T cells.

Single cell-derived clones were obtained for allele analysis. The frequencies of C/G-to-non-C/G edits in each single cell-derived clone were determined with targeted deep sequencing. **a,** Single cell-derived clones from a population of HEK 293T cells treated with *ND1*-targeted mitoZFD. **b,** Single cell-derived clones from a population of HEK 293T cells treated with *ND2*-targeted mitoZFD. **c,** Single cell-derived clones from a population of untreated HEK 293T cells. The ZFP binding sites are shown in green. The high editing frequencies in clones that underwent mitoZFD-induced editing are shown in red.

Human *MT-ND1*	Ref.	GTTTACTCAATCCTCTGATCAGGGTGAGCATCA	(H-strand)	
**Sample**			**AA change**	**Read (%)**
Clone 3<br>: 34 %	Allele-1	GTTTACTCAATCCTCTGATCAGGGTGAGCATCA	-	62
		V Y S I L W S G W A S		
	Allele-2	GTTTACTCAATCCTCTGATtAGGGTGAGCATCA	S119L	21
		V Y S I L W L G W A S		
	Allele-3	GTTTACTCAATCCTCTaATCAGGGTGAGCATCA	W118*	13
		V Y S I L * S G W A S		
Clone 10<br>: 92%	Allele-1	GTTTACTCAATCCTCTGATtAGGGTGAGCATCA	S119L	40
		V Y S I L W L G W A S		
	Allele-2	GTTTACTCAATCCTCTaATCAGGGTGAGCATCA	W118*	32
		V Y S I L * S G W A S		
	Allele-3	GTTTACTCAATCCTCTaATtAGGGTGAGCATCA	W118*, S119L	20
		V Y S I L * L G W A S		
	Allele-4	GTTTACTCAATCCTCTGATCAGGGTGAGCATCA	-	2
		V Y S I L W S G W A S		
Clone 16<br>: 80%	Allele-1	GTTTACTCAATCCTCTGATtAGGGTGAGCATCA	S119L	40
		V Y S I L W L G W A S		
	Allele-2	GTTTACTCAATCCTCTaATCAGGGTGAGCATCA	W118*	35
		V Y S I L * S G W A S		
	Allele-3	GTTTACTCAATCCTCTGATCAGGGTGAGCATCA	-	16
		V Y S I L W S G W A S		
	Allele-4	GTTTACTCAATCCTCTaATtAGGGTGAGCATCA	W118*, S119L	5
		V Y S I L * L G W A S		
Clone 27<br>: 45%	Allele-1	GTTTACTCAATCCTCTGATCAGGGTGAGCATCA	-	50
		V Y S I L W S G W A S		
	Allele-2	GTTTACTCAATCCTCTGATtAGGGTGAGCATCA	S119L	26
		V Y S I L W L G W A S		
	Allele-3	GTTTACTCAATCCTCTaATCAGGGTGAGCATCA	W118*	19
		V Y S I L * S G W A S		
Clone 30<br>: 90%	Allele-1	GTTTACTCAATCCTCTGATtAGGGTGAGCATCA	S119L	40
		V Y S I L W L G W A S		
	Allele-2	GTTTACTCAATCCTCTaATCAGGGTGAGCATCA	W118*	35
		V Y S I L * S G W A S		
	Allele-3	GTTTACTCAATCCTCTaATtAGGGTGAGCATCA	W118*, S119L	15
		V Y S I L * L G W A S		
	Allele-4	GTTTACTCAATCCTCTGATCAGGGTGAGCATCA	-	5
		V Y S I L W S G W A S		

Supplementary figure 9. Analysis of allele frequencies in single cell-derived clones from a population of *ND1*-mitoZFD-treated HEK 293T cells.
Allele analysis in single cell-derived clones that exhibited high frequencies of base editing. The table shows the amino acids that were changed as a result of base editing in *ND1*. In the reference sequence at the top, the red letters indicate the spacer. In the alleles, the red letters indicate changes in the amino acid sequence. (* indicates a stop codon.)

Human *MT-ND2*	Ref.	CTACGCCTAATCTACTCCACCTCAATCACACTACTCCCC	(H-strand)	
**Sample**			**AA change**	**Read (%)**
Clone 21	Allele-1	CTACGCCTAATCTACTttACCTCAATCACACTACTCCCC<br>L R L I Y F T S I T L L P	S299F	45
	Allele-2	CTACGCCTAATCTACTCCACCTCAATCACACTACTCCCC<br>L R L I Y S T S I T L L P	-	31
	Allele-3	CTACGCCTAATCTACTttAttTCAATCACACTACTCCCC<br>L R L I Y F I S I T L L P	S299F, T300I	10
	Allele-4	CTACGCCTAATCTACTtCACCTCAATCACACTACTCCCC<br>L R L I Y F T S I T L L P	S299F	4
	Allele-5	CTACGCCTAATCTACTttAtCTCAATCACACTACTCCCC<br>L R L I Y F I S I T L L P	S299F, T300I	3
	Allele-6	CTACGCCTAATCTACTttACCTtAATCACACTACTCCCC<br>L R L I Y F T L I T L L P	S299F, S301L	1
Clone 23	Allele-1	CTACGCCTAATCTACTttACCTCAATCACACTACTCCCC<br>L R L I Y F T S I T L L P	S299F	43
	Allele-2	CTACGCCTAATCTACTCCACCTCAATCACACTACTCCCC<br>L R L I Y S T S I T L L P	-	27
	Allele-3	CTACGCCTAATCTACTttAttTCAATCACACTACTCCCC<br>L R L I Y F I S I T L L P	S299F, T300I	16
	Allele-4	CTACGCCTAATCTACTttAtCTCAATCACACTACTCCCC<br>L R L I Y F I S I T L L P	S299F, T300I	6
	Allele-5	CTACGCCTAATCTACTttACCTtAATCACACTACTCCCC<br>L R L I Y F T L I T L L P	S299F, S301L	1
Clone 24	Allele-1	CTACGCCTAATCTACTttACCTCAATCACACTACTCCCC<br>L R L I Y F T S I T L L P	S299F	34
	Allele-2	CTACGCCTAATCTACTttAttTCAATCACACTACTCCCC<br>L R L I Y F I S I T L L P	S299F, T300I	30
	Allele-3	CTACGCCTAATCTACTCCACCTCAATCACACTACTCCCC<br>L R L I Y S T S I T L L P	-	23
	Allele-4	CTACGCCTAATCTACTttAtCTCAATCACACTACTCCCC<br>L R L I Y F I S I T L L P	S299F, T300I	3
	Allele-5	CTACGCCTAATCTACTttAttTtAATCACACTACTCCCC<br>L R L I Y F I L I T L L P	S299F, T300I, S301L	1
	Allele-6	CTACGCCTAATCTACTtCACCTCAATCACACTACTCCCC<br>L R L I Y F T S I T L L P	S299F	1
Clone 31	Allele-1	CTACGCCTAATCTACTCCACCTCAATCACACTACTCCCC<br>L R L I Y S T S I T L L P	-	53
	Allele-2	CTACGCCTAATCTACTttACCTCAATCACACTACTCCCC<br>L R L I Y F T S I T L L P	S299F	28
	Allele-3	CTACGCCTAATCTACTttAttTCAATCACACTACTCCCC<br>L R L I Y F I S I T L L P	S299F, T300I	9
	Allele-4	CTACGCCTAATCTACTttAtCTCAATCACACTACTCCCC<br>L R L I Y F I S I T L L P	S299F, T300I	2
	Allele-5	CTACGCCTAATCTACTtCACCTCAATCACACTACTCCCC<br>L R L I Y F T S I T L L P	S299F	2
Clone 32	Allele-1	CTACGCCTAATCTACTCCACCTCAATCACACTACTCCCC<br>L R L I Y S T S I T L L P	-	40
	Allele-2	CTACGCCTAATCTACTttACCTCAATCACACTACTCCCC<br>L R L I Y F T S I T L L P	S299F	37
	Allele-3	CTACGCCTAATCTACTttAttTCAATCACACTACTCCCC<br>L R L I Y F I S I T L L P	S299F, T300I	11
	Allele-4	CTACGCCTAATCTACTttAtCTCAATCACACTACTCCCC<br>L R L I Y F I S I T L L P	S299F, T300I	3
	Allele-5	CTACGCCTAATCTACTtCACCTCAATCACACTACTCCCC<br>L R L I Y F T S I T L L P	S299F	2
Clone 33	Allele-1	CTACGCCTAATCTACTCCACCTCAATCACACTACTCCCC<br>L R L I Y S T S I T L L P	-	46
	Allele-2	CTACGCCTAATCTACTttACCTCAATCACACTACTCCCC<br>L R L I Y F T S I T L L P	S299F	34
	Allele-3	CTACGCCTAATCTACTttAttTCAATCACACTACTCCCC<br>L R L I Y F I S I T L L P	S299F, T300I	12
	Allele-4	CTACGCCTAATCTACTttAtCTCAATCACACTACTCCCC<br>L R L I Y F I S I T L L P	S299F, T300I	2
	Allele-5	CTACGCCTAATCTACTtCACCTCAATCACACTACTCCCC<br>L R L I Y F T S I T L L P	S299F	1
Clone 34	Allele-1	CTACGCCTAATCTACTCCACCTCAATCACACTACTCCCC<br>L R L I Y S T S I T L L P	-	72
	Allele-2	CTACGCCTAATCTACTttACCTCAATCACACTACTCCCC<br>L R L I Y F T S I T L L P	S299F	18
	Allele-3	CTACGCCTAATCTACTttAttTCAATCACACTACTCCCC<br>L R L I Y F I S I T L L P	S299F, T300I	3
	Allele-4	CTACGCCTAATCTACTttAtCTCAATCACACTACTCCCC<br>L R L I Y F I S I T L L P	S299F, T300I	1
	Allele-5	CTACGCCTAATCTACTtCACCTCAATCACACTACTCCCC<br>L R L I Y F T S I T L L P	S299F	0.4

Supplementary figure 10. Analysis of allele frequencies in single cell-derived clones from a population of *ND2*-mitoZFD-treated HEK 293T cells.

Allele analysis in single cell-derived clones that exhibited high frequencies of base editing. The table shows the amino acids that were changed as a result of base editing in *ND2*. In the reference sequence at the top, the red letters indicate the spacer. In the alleles, the red letters indicate changes in the amino acid sequence.

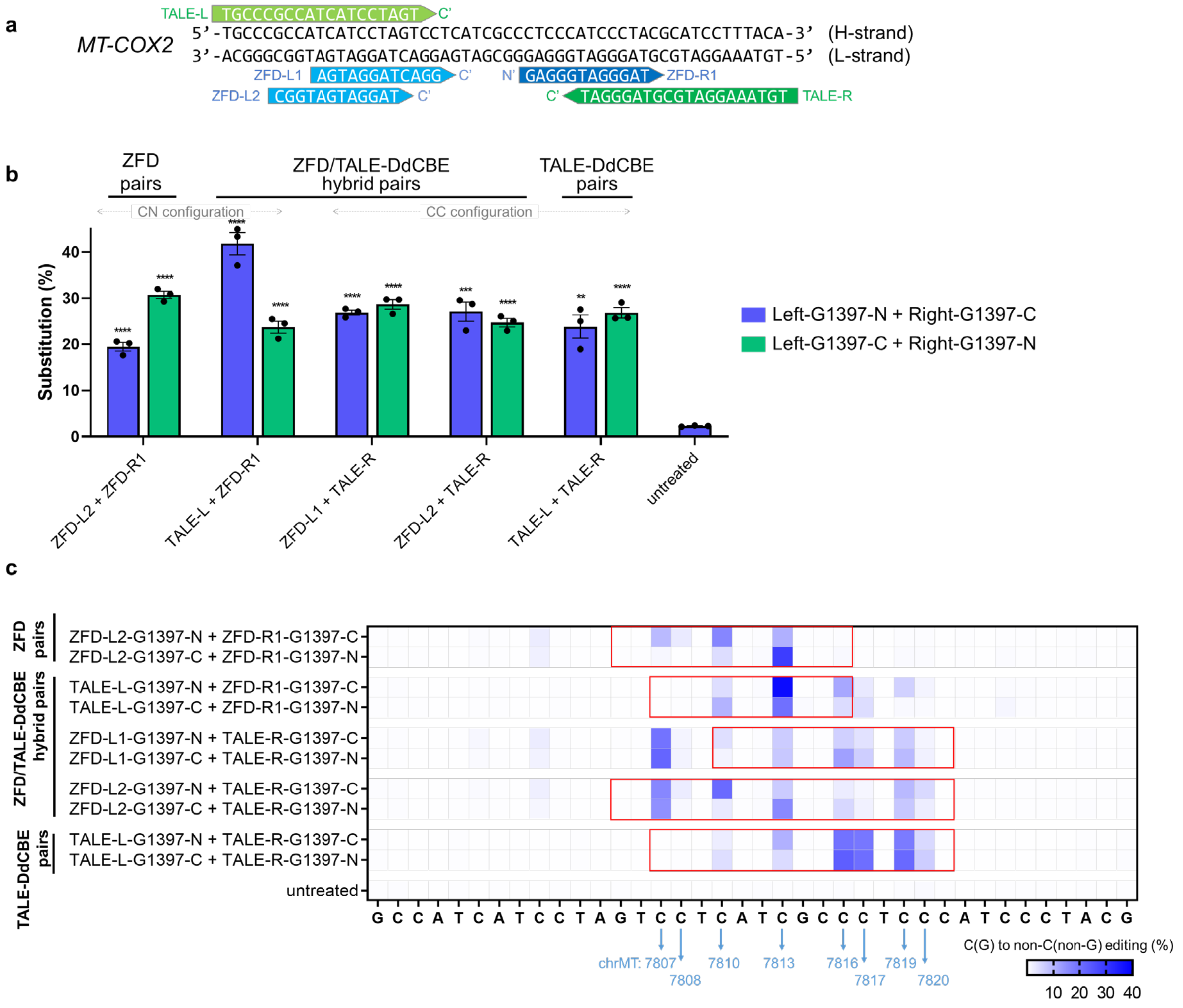

Supplementary figure 11. Base editing activities of mitoZFDs, TALE-based DdCBEs, and ZFD/DdCBE hybrid pairs for the *Cox2* site.

a, DNA sequences of the binding regions of the mitoZFD and TALE-DdCBE pairs (Supplementary Table 9) for the *Cox2* site. Sites recognized by the TALE-DdCBEs are shown in green and for the mitoZFDs in blue. The upper sequence represents the mtDNA heavy strand and the lower sequence represents the mtDNA light strand. **b,** Frequencies of cytosines edited by ZFDs, TALE-DdCBEs, and ZFD/DdCBE hybrid pairs obtained using targeted deep sequencing. All statistical analysis for comparing with untreated samples was conducted using unpaired Student's t-test (two-tailed) in GraphPad Prism 8. Statistical significance as compared with untreated samples was denoted with * = $p \leq 0.05$, ** = $p \leq 0.01$, *** = $p \leq 0.001$, **** = $p \leq 0.0001$, n.s. (not significant) = $p > 0.05$. p values of Left-G1397-N + Right-G1397-C form of ZFD-L2 + ZFD-R1, TALE-L + ZFD-R1, ZFD-L1 + TALE-R, ZFD-L2 + TALE-R, and TALE + TALE-R are 0.000052, 0.000079, 0.000001, 0.000269 and 0.001124. p values of Left-G1397-C + Right-G1397-N form of ZFD-L2 + ZFD-R1, TALE-L + ZFD-R1, ZFD-L1 + TALE-R, ZFD-L2 + TALE-R, and TALE + TALE-R are 0.000004, 0.000081, 0.000014, 0.000017 and 0.000026. Data are shown as means with standard error of the mean (s.e.m.) from n=3 biologically independent samples. **c,** Heat maps of base editing activities at each base position. The red box indicates the spacer region. The blue arrows indicate the position in the mtDNA. Source data are provided as a Source Data file.

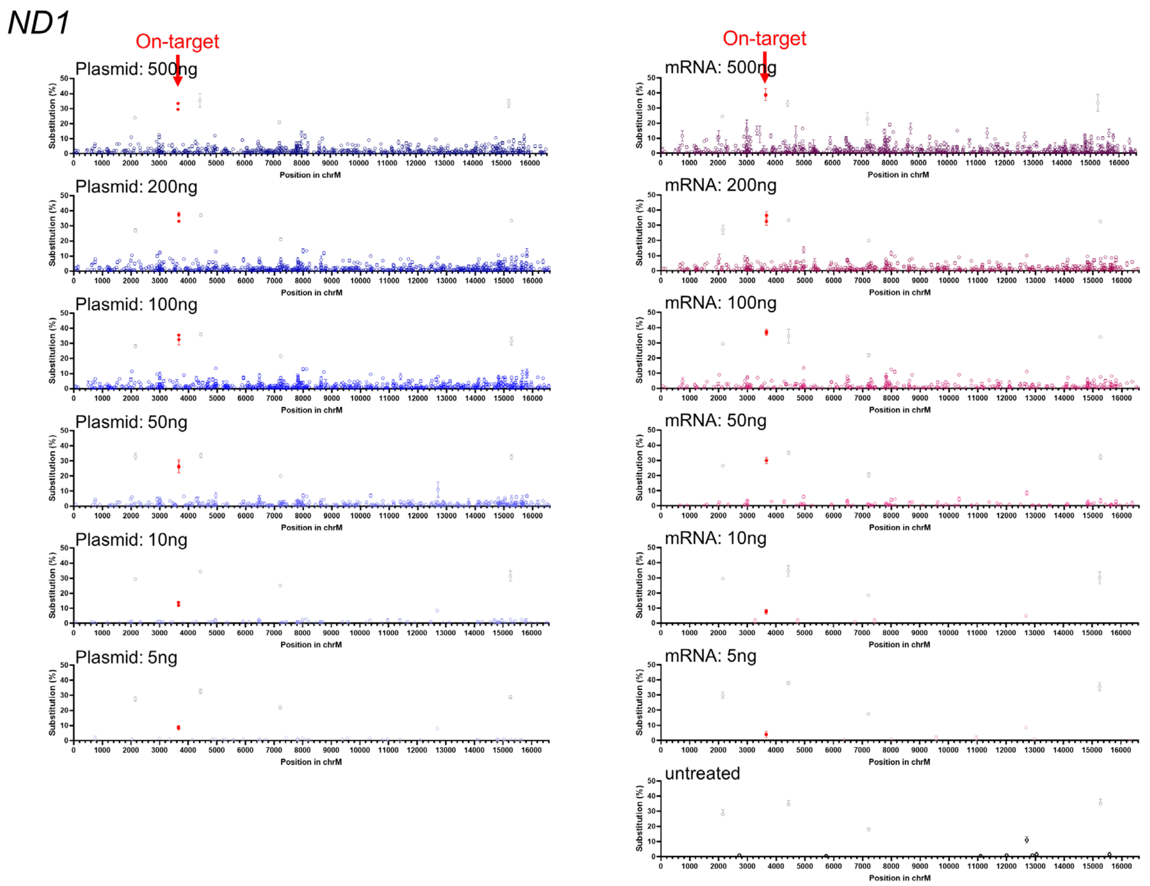

Supplementary figure 12. Mitochondrial genome-wide target specificity of the *ND1*-targeted mitoZFD depends on the concentration of the ZFD-encoding mRNA or plasmid. On- and off-target editing frequencies determined by whole-mtDNA sequencing. Results from HEK 293T cells transfected with the indicated amounts of *ND1*-targeted mitoZFD-encoding plasmid or mRNA are shown on the plots. The red arrows indicate the on-target site, the red dots indicate the frequency of base editing at the on-target site, and gray dots indicate SNPs that are also present in controls. Error bars are the standard error of the mean (s.e.m.) for n = 2 biologically independent samples. Source data are provided as a Source Data file.

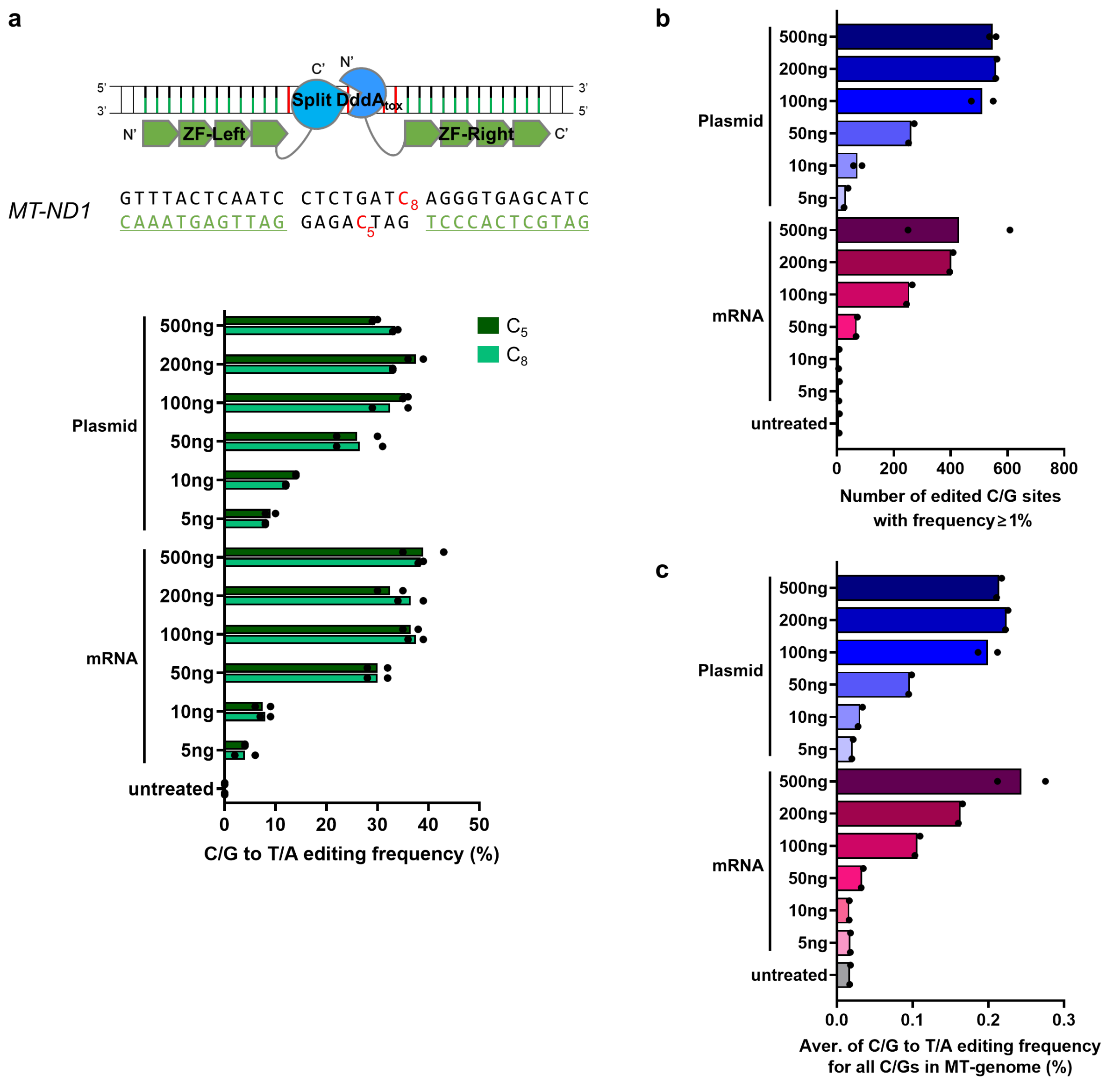

Supplementary figure 13. Analysis of on- and off-target activity of *ND1*-targeted mitoZFD.

a, The illustration on the top shows the ZFD binding at the *ND1* site. The ZFD binding sites are indicated in green. The on-target cytosines in the spacer are indicated in red. On-target activity determined from whole-mtDNA sequencing data from Supplementary figure 12. The activity decreases as the amount of transfected mitoZFD-encoding plasmid or mRNA decreases. **b,** The number of C/G sites that are edited with a frequency > 1% for each amount of plasmid or mRNA. **c,** The average C/G-to-T/A editing frequency for all C/Gs in the mitochondrial genome for each amount of plasmid or mRNA. Data are shown as means from n = 2 biologically independent samples. Source data are provided as a Source Data file.

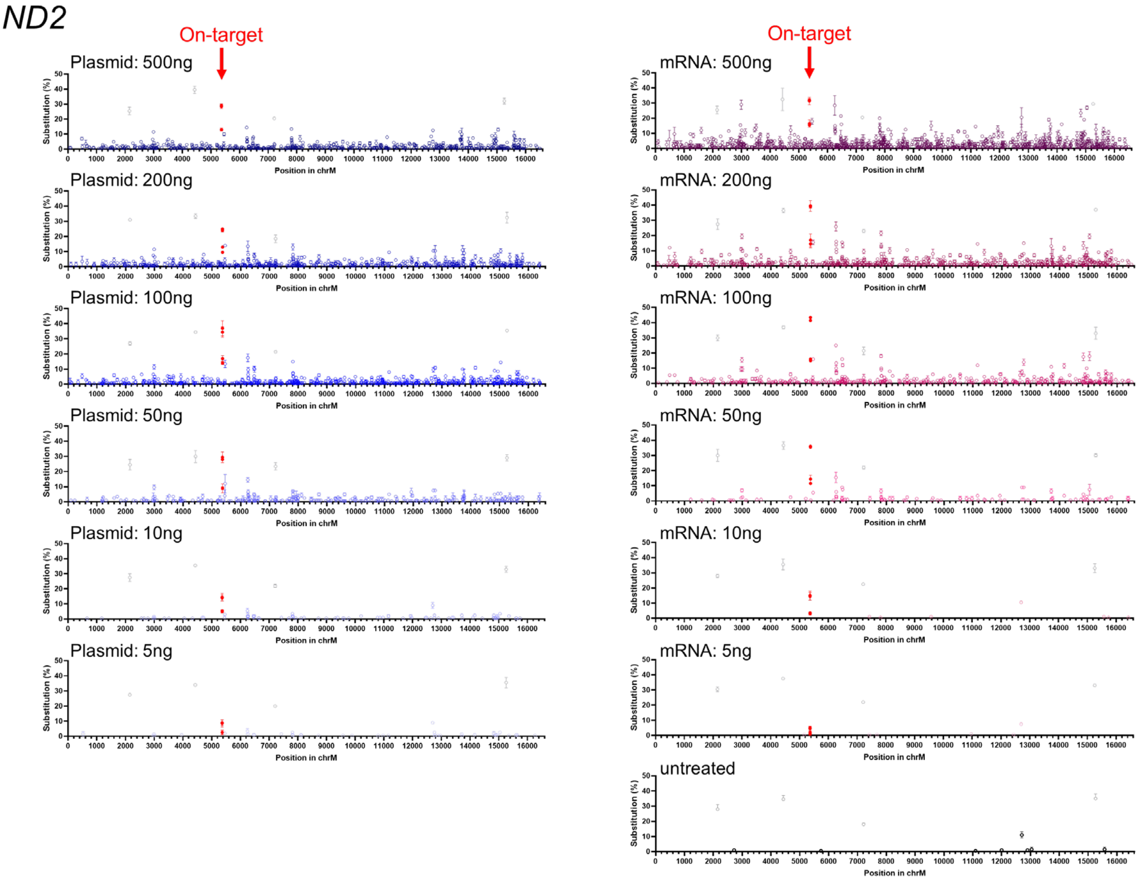

Supplementary figure 14. Mitochondrial genome-wide target specificity of the *ND2*-targeted mitoZFD depends on the concentration of the ZFD-encoding mRNA or plasmid. On- and off-target editing frequencies determined by whole-mtDNA sequencing. Results from HEK 293T cells transfected with the indicated amounts of *ND2*-targeted mitoZFD-encoding plasmid or mRNA are shown on the plots. The red arrows indicate the on-target site, the red dotes indicate the frequency of base editing at the on-target site, and gray dots indicate SNPs that are also present in controls. Error bars are the standard error of the mean (s.e.m.) for n = 2 biologically independent samples. Source data are provided as a Source Data file.

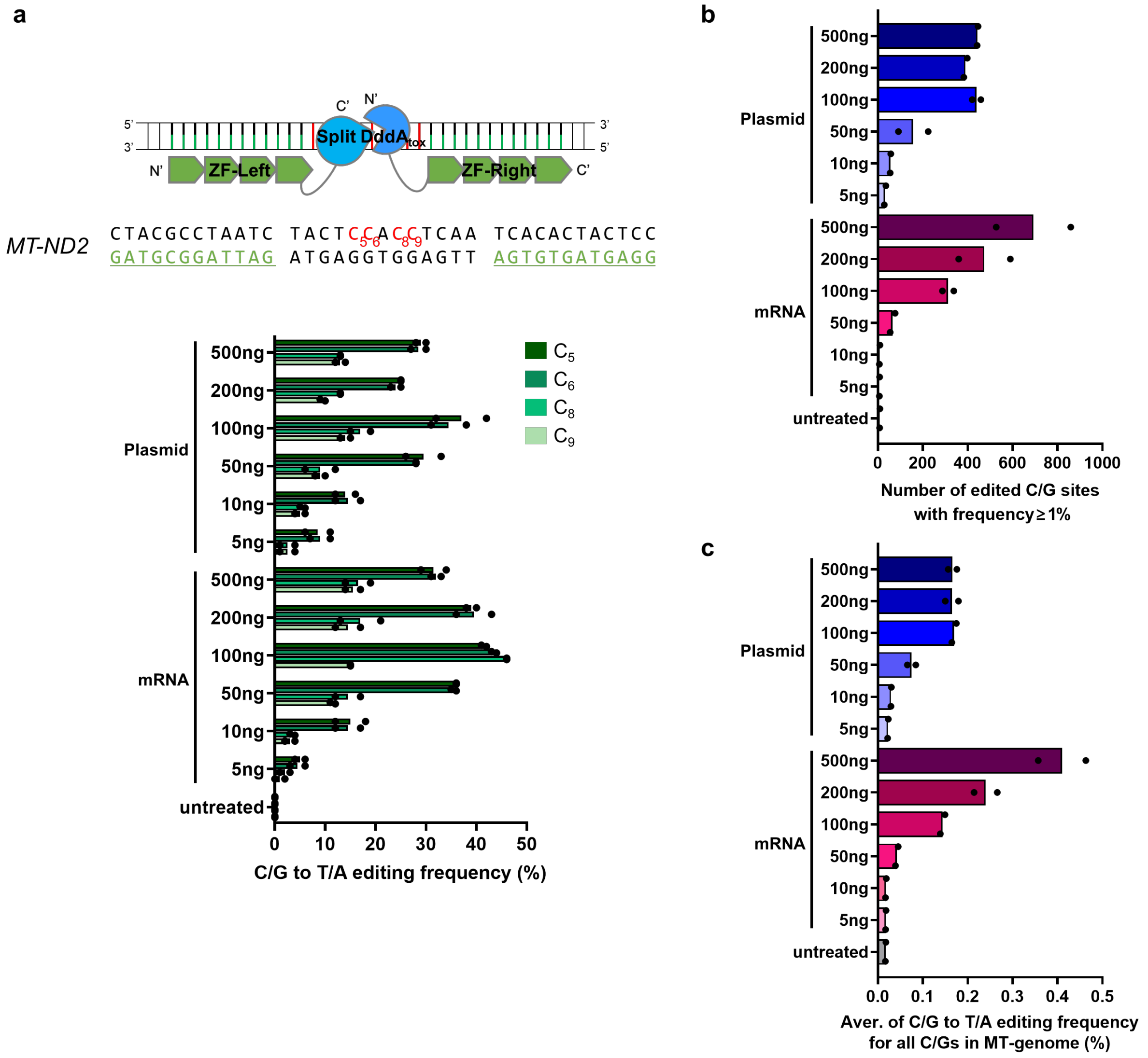

Supplementary figure 15. Analysis of the on- and off-target activity of *ND2*-targeted mitoZFD.

a, The illustration on the top shows the ZFD binding at the *ND2* site. The ZFD binding sites are indicated in green. The on-target cytosines in the spacer are indicated in red. On-target activity determined from whole-mtDNA sequencing data from Supplementary figure 14. The activity decreases as the amount of transfected mitoZFD-encoding plasmid or mRNA decreases. **b,** The number of C/G sites that are edited with a frequency > 1% for each amount of plasmid or mRNA. **c,** The average C/G-to-T/A editing frequency for all C/Gs in the mitochondrial genome for each amount of plasmid or mRNA. Data are shown as means from n = 2 biologically independent samples. Source data are provided as a Source Data file.

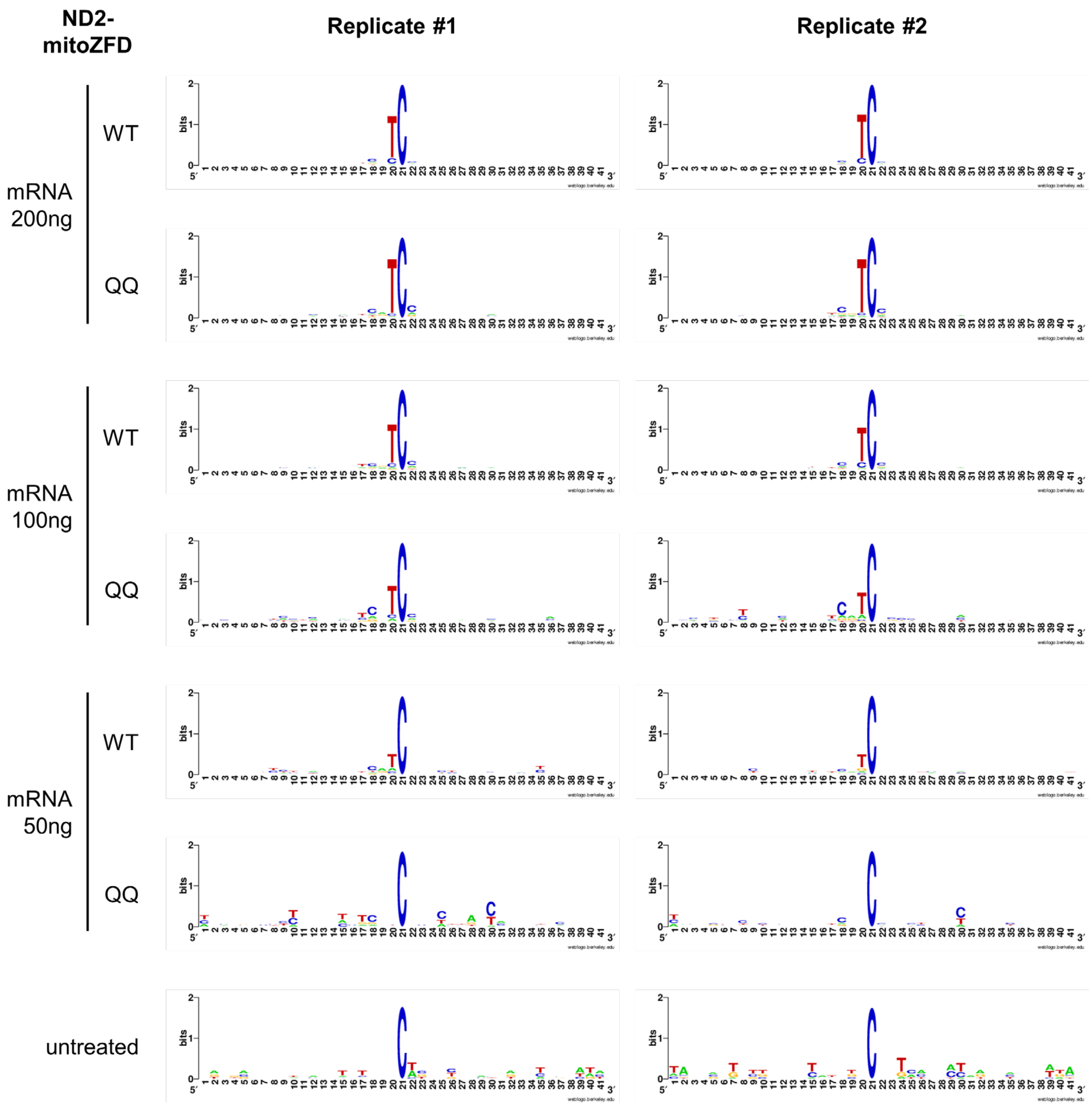

Supplementary figure 16. Sequence logos with off-target sites of *ND2*-targeted mitoZFD. Sequence logos obtained via WebLogo using DNA sequences of the region flanking mutated cytosines from Fig. 5.

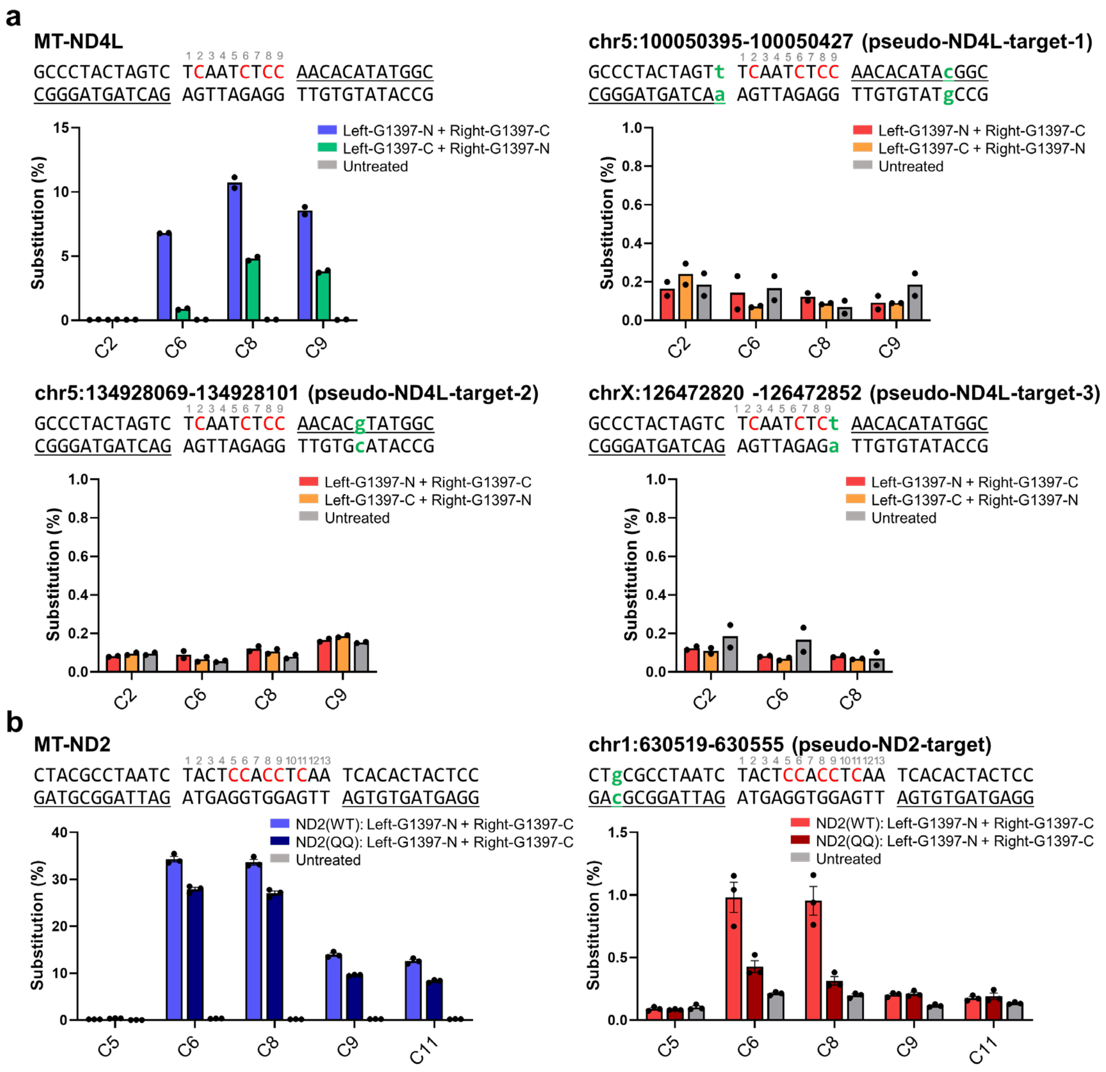

Supplementary figure 17. Potential off-target sites of mitoZFD in nuclear DNA.
Base editing activity at each base position within the spacer at the on-target and potential off-target sites in nuclear DNA with high sequence homology for the *ND4L* **(a)** and *ND2* **(b)** target. ZFP-binding sites are underlined and mismatches bases are shown in green. Data are shown as means from n = 2 biologically independent samples (a) or means with the standard error of the mean (s.e.m.) for n = 3 biologically independent samples (b). Source data are provided as a Source Data file.

Supplementary table 1. Spacer sequences, ranging from 1-24 bps in length, in the pTarget plasmids.

Spacer length (bp)	Left-ZFD DNA binding region	Spacer sequence	Right-ZFD DNA binding region
1	GTCATCCTCATC	C	AAACTGCAAAAG
2	GTCATCCTCATC	TC	AAACTGCAAAAG
3	GTCATCCTCATC	TCG	AAACTGCAAAAG
4	GTCATCCTCATC	CTGA	AAACTGCAAAAG
5	GTCATCCTCATC	CTGAT	AAACTGCAAAAG
6	GTCATCCTCATC	CTGATC	AAACTGCAAAAG
7	GTCATCCTCATC	CTGATCG	AAACTGCAAAAG
8	GTCATCCTCATC	CTGATCGA	AAACTGCAAAAG
9	GTCATCCTCATC	CTGATCGAT	AAACTGCAAAAG
10	GTCATCCTCATC	CTGATCGATC	AAACTGCAAAAG
11	GTCATCCTCATC	CTGATCGATCG	AAACTGCAAAAG
12	GTCATCCTCATC	CTGATCGATCGA	AAACTGCAAAAG
13	GTCATCCTCATC	CTGATCGATCGAT	AAACTGCAAAAG
14	GTCATCCTCATC	CTGATCGATCGATC	AAACTGCAAAAG
15	GTCATCCTCATC	CTGATCGATCGATCG	AAACTGCAAAAG
16	GTCATCCTCATC	CTGATCGATCGATCGA	AAACTGCAAAAG
17	GTCATCCTCATC	CTGATCGATCGATCGAT	AAACTGCAAAAG
18	GTCATCCTCATC	CTGATCGATCGATCGATC	AAACTGCAAAAG
19	GTCATCCTCATC	CTGATCGATCGATCGATCG	AAACTGCAAAAG
20	GTCATCCTCATC	CTGATCGATCGATCGATCGA	AAACTGCAAAAG
21	GTCATCCTCATC	CTGATCGATCGATCGATCGAT	AAACTGCAAAAG
22	GTCATCCTCATC	CTGATCGATCGATCGATCGATC	AAACTGCAAAAG
23	GTCATCCTCATC	CTGATCGATCGATCGATCGATCG	AAACTGCAAAAG
24	GTCATCCTCATC	CTGATCGATCGATCGATCGATCGA	AAACTGCAAAAG

Supplementary table 2. Amino acid sequences of ZFD constructs used for the optimization of the ZFD architecture.

- Left-ZFD: SV40 NLS–ZFP(S162-left)–linker–DddA$_{tox}$ half–4aa linker–UGI
- Right-ZFD: SV40 NLS–ZFP(S162-right)–linker–DddA$_{tox}$ half–4aa linker–UGI

- SV40 NLS:<br>PKKKRKV
- ZFP(S162-left)<br>GIHGVPAAMAERPFQCRICMRNFSDRSNLSRHIRTHTGEKPFACDICGRKFAISSNL<br>NSHTKIHTGSQKPFQCRICMRNFSRSDNLARHIRTHTGEKPFACDICGRKFATSGNL<br>TRHTKIHLR<br><br>- ZFP(S162-right)<br>GIHGVPAAMAERPFQCRICMRNFSRSDNLSVHIRTHTGEKPFACDICGRKFAQKIN<br>LQVHTKIHTGEKPFQCRICMRNFSRSDVLSEHIRTHTGEKPFACDICGRKFAQRNH<br>RTTHTKIHLR
- Linker between Zinc finger protein and DddA$_{tox}$ half:<br>- 2aa: GS<br>- 5aa: TGEKP<br>- 10aa: SGAQGSTLDF<br>- 16aa: SGSETPGTSESATPES<br>- 24aa: SGTPHEVGVYTLSGTPHEVGVYTL<br>- 32aa: SGGSSGGSSGSETPGTSESATPESSGGSSGGS
- Split-DddA$_{tox}$ G1333-N<br>GSYALGPYQISAPQLPAYNGQTVGTFYYVNDAGGLESKVFSSGG<br><br>- Split-DddA$_{tox}$ G1333-C<br>PTPYPNYANAGHVEGQSALFMRDNGISEGLVFHNNPEGTCGFCVNMTETLLPENA<br>KMTVVPPEGAIPV KRGATGETKVFTGNSNSPKSPTKGGC
- Split-DddA$_{tox}$ G1397-N<br>GSYALGPYQISAPQLPAYNGQTVGTFYYVNDAGGLESKVFSSGGPTPYPNYANAG<br>HVEGQSALFMRDN GISEGLVFHNNPEGTCGFCVNMTETLLPENAKMTVVPPEG<br><br>- Split-DddA$_{tox}$ G1397-C<br>AIPVKRGATGETKVFTGNSNSPKSPTKGGC
- 4aa linker<br>SGGS
- UGI<br>TNLSDIIEKETGKQLVIQESILMLPEEVEEVIGNKPESDILVHTAYDESTDENVMLLT<br>SDAPEYKPWA LVIQDSNGENKIKML

Supplementary table 3. Amino acid sequences of ZFD constructs for endogenous target sites.

- C type: SV40 NLS–Zinc finger protein–24aa linker–DddA$_{tox}$ half–4aa linker–UGI
- N type: SV40 NLS–DddA$_{tox}$ half–24aa linker–Zinc finger protein–4aa linker–UGI

- SV40 NLS<br>PKKKRKV
- 24aa linker<br>SGTPHEVGVYTLSGTPHEVGVYTL
- Split-DddA$_{tox}$ G1397-N<br>GSYALGPYQISAPQLPAYNGQTVGTFYYVNDAGGLESKVFSSGGPTPYPNYANAG<br>HVEGQSALFMRDN GISEGLVFHNNPEGTCGFCVNMTETLLPENAKMTVVPPEG<br><br>- Split-DddA$_{tox}$ G1397-C<br>AIPVKRGATGETKVFTGNSNSPKSPTKGGC
- 4aa linker<br>SGGS
- UGI<br>TNLSDIIEKETGKQLVIQESILMLPEEVEEVIGNKPESDILVHTAYDESTDENVMLLT<br>SDAPEYKPWA LVIQDSNGENKIKML
- ZFP<br><br>CCR5-1 Left (C type) [S162 ZFN-Left]<br>GIHGVPAAMAERPFQCRICMRNFSDRSNLSRHIRTHTGEKPFACDICGRKFAISSNL<br>NSHTKIHTGSQKPFQCRICMRNFSRSDNLARHIRTHTGEKPFACDICGRKFATSGNL<br>TRHTKIHLR<br><br>CCR5-1 Right (C type) [S162 ZFN-Right]<br>GIHGVPAAMAERPFQCRICMRNFSRSDNLSVHIRTHTGEKPFACDICGRKFAQKIN<br>LQVHTKIHTGEKPFQCRICMRNFSRSDVLSEHIRTHTGEKPFACDICGRKFAQRNH<br>RTTHTKIHLR<br><br>CCR5-2 Left (C type) [S162 ZFN-Left]<br>GIHGVPAAMAERPFQCRICMRNFSDRSNLSRHIRTHTGEKPFACDICGRKFAISSNL<br>NSHTKIHTGSQKPFQCRICMRNFSRSDNLARHIRTHTGEKPFACDICGRKFATSGNL<br>TRHTKIHLR<br><br>CCR5-2 Right (C type) [Modifying S162 ZFN-Right with additional ZF using the Barbas set of zinc finger modules]<br>GIHGVPAAMAERPFQCRICMRNFSQSGDLRRHIRTHTGEKPFACDICGRKFARSDN<br>LSVHTKIHTGSQKPFQCRICMRNFSQKINLQVHIRTHTGEKPFACDICGRKFARSDV<br>LSEHTKIHLR<br><br>TRAC-Left (C type) [Adapted from Paschon, D.E. et al., 2019]<br>GIHGVPAAMAERPFQCRICMRNFSDQSNLRAHIRTHTGEKPFACDICGRKFATSSN<br>RKTHTKIHTGSQKPFQCRICMRNFSLQQTLADHIRTHTGEKPFACDICGRKFAQSG<br>NLARHTKIHLR

TRAC-Left (N type) [Adapted from Paschon, D.E. et al., 2019]
FQCRICMRKFATSGSLTRHTKIHTGEKPFQCRICMRNFSRSDHLSTHIRTHTGEKPF
ACDICGRKFATSSNRTKHTKIHTHPRAPIPKPFQCRICMRNFSRSDNLSEHIRTHTGE
KPFACDICGRKFAWHSSLRVHTKIHLR

TRAC-Right (C type) [From Paschon, D.E. et al., 2019]
GIHGVPAAMAERPFQCRICMRNFSRSDHLSTHIRTHTGEKPFACDICGRKFADRSH
LARHTKIHTGSQKPFQCRICMRKFALKQHLNEHTKIHTGEKPFQCRICMRNFSQSG
NLARHIRTHTGEKPFACDICGRKFAHNSSLKDHTKIHLR

MFAP1 Left (C type) [De novo designed using the Toolgen set of zinc finger modules]
GIRIPGEKPYSCGICGKSFSDSSAKRRHCILHTGEKPYTCSDCGKAFRDKSCLNRHR
RTHTGEKPYKCMECGKAFNRRSHLTRHQRIHTGEKPYECNYCGKTFSVSSTLIRHQ
RIHLR

MFAP1 Right (C type) [Modifying S162 ZFN-Right with additional ZF using the Barbas set of zinc finger modules]
GIRERPYACPVESCDRRFSTSGSLVRHIRIHTGQKPFQCRICMRNFSRSDELTRHIRT
HTGEKPFACDICGRKFARSDHLTTHTKIHTGEKPFQCRICMRKFAQSSNLVRHTKI
HLR

CCDC28B Left (C type) [Modifying S162 ZFN-Right with additional ZF using the Barbas set of zinc finger modules]
GIRERPYACPVESCDRRFSDPGHLVRHIRIHTGQKPFQCRICMRNFSRSDELTRHIRT
HTGEKPFACDICGRKFARSDHLTTHTKIHTGEKPFQCRICMRKFARSDKLVRHTKI
HLR

CCDC28B Right (C type) [De novo designed using the Toolgen set of zinc finger modules]
GIRIPGEKPYECNYCGKTFSVSSTLIRHQRIHTGEKPYHCDWDGCGWKFARSDELT
RHYRKHTGEKPFECKDCGKAFIQKSNLIRHQRTHTGEKPYHCDWDGCGWKFARS
DELTRHYRKHLR

KDM4B Left (C type) [Modifying S162 ZFN-Right with additional ZF using the Barbas set of zinc finger modules]
GIRERPYACPVESCDRRFSDCRDLARHIRIHTGQKPFQCRICMRNFSRSDELTRHIRT
HTGEKPFACDICGRKFARSDHLTTHTKIHTGEKPFQCRICMRKFARSDKLVRHTKI
HLR

KDM4B Right (C type) [De novo designed using the Toolgen set of zinc finger modules]
GIRIPGEKPFECKDCGKAFIQKSNLIRHQRTHTGEKPYRCEECGKAFRWPSNLTRH
KRIHTGEKPYRCEECGKAFRWPSNLTRHKRIHTGEKPYSCGICGKSFSDSSAKRRH
CILHLR

NUMBL Left (C type) [Modifying S162 ZFN-Right with additional ZF using the Barbas set of zinc finger modules]
GIRERPYACPVESCDRRFSDCRDLARHIRIHTGQKPFQCRICMRNFSRSDELTRHIRT
HTGEKPFACDICGRKFARSDHLTTHTKIHTGEKPFQCRICMRKFARSDKLVRHTKI
HLR

NUMBL Right (C type) [De novo designed using the Toolgen set of zinc finger modules]

GIRIPGEKPYTCSDCGKAFRDKSCLNRHRRTHTGEKPYKCGQCGKFYSQVSHLTR
HQKIHTGEKPFECKDCGKAFIQKSNLIRHQRTHTGEKPYHCDWDGCGWKFARSDE
LTRHYRKHLR

INPP5D-1 Left (C type) [Modifying S162 ZFN-Right with additional ZF using the Barbas set of zinc finger modules]
GIRERPYACPVESCDRRFSRSDKLVRHIRIHTGQKPFQCRICMRNFSRSDELTRHIRT
HTGEKPFACDICGRKFARSDHLTTHTKIHTGEKPFQCRICMRKFARSDKLVRHTKI
HLR

INPP5D-1 Right (C type) [De novo designed using the Toolgen set of zinc finger modules]
GIRIPGEKPYTCSDCGKAFRDKSCLNRHRRTHTGEKPYECNYCGKTFSVSSTLIRHQ
RIHTGEKPYTCSDCGKAFRDKSCLNRHRRTHTGEKPYTCSDCGKAFRDKSCLNRH
RRTHLR

INPP5D-2 Left (C type) [Modifying S162 ZFN-Right with additional ZF using the Barbas set of zinc finger modules]
GIRERPYACPVESCDRRFSRSDKLVRHIRIHTGQKPFQCRICMRNFSRSDELTRHIRT
HTGEKPFACDICGRKFARSDHLTTHTKIHTGEKPFQCRICMRKFARSDKLVRHTKI
HLR

INPP5D-2 Right (C type) [De novo designed using the Toolgen set of zinc finger modules]
GIRIPGEKPYECNYCGKTFSVSSTLIRHQRIHTGEKPYTCSDCGKAFRDKSCLNRHR
RTHTGEKPYTCSDCGKAFRDKSCLNRHRRTHTGEKPYHCDWDGCGWKFARSDEL
TRHYRKH

DVL3 Left (C type) [De novo designed using Barbas zinc finger modules]
GIHGVPAAMAERPFQCRICMRNFSTSGHLVRHIRTHTGEKPFACDICGRKFATSGH
LVRHTKIHTGEKPFQCRICMRNFSTSGELVRHIRTHTGEKPFACDICGRKFAQSSNL
VRHTKIHLR

DVL3 Right (C type) [S162 ZFN-left]
GIHGVPAAMAERPFQCRICMRNFSDRSNLSRHIRTHTGEKPFACDICGRKFAISSNL
NSHTKIHTGSQKPFQCRICMRNFSRSDNLARHIRTHTGEKPFACDICGRKFATSGNL
TRHTKIHLR

Supplementary table 4. DNA sequences of zinc finger binding sites and spacer region of ZFDs for endogenous target sites.
The ZFD binding sites are indicated in green.

		Left-ZFD binding region	Spacer region	Right-ZFD binding region		ZFD configuration
MFAP1	5'-	GACGGCCCCAGC	CGTAGACT	GAATGGGCGGTT	-3'	C-C
	3'-	CTGCCGGGGTCG	GCATCTGA	CTTACCCGCCAA	-5'	
CCDC28B	5'-	TGCCGCCCAGTC	CTGGCTGGAG	GTGGAAGCGGCT	-3'	C-C
	3'-	ACGGCGGGTCAG	GACCGACCTC	CACCTTCGCCGA	-5'	
KDM4B	5'-	GGCCGCCCACCC	GGTCCCCACT	GTCGGTGGTGAA	-3'	C-C
	3'-	CCGGCGGGTGGG	CCAGGGGTGA	CAGCCACCACTT	-5'	
NUMBL	5'-	GGCCGCCCACCC	CCAGACAG	GCGGAAGGAGCC	-3'	C-C
	3'-	CCGGCGGGTGGG	GGTCTGTC	CGCCTTCCTCGG	-5'	
INPP5D-1	5'-	CCCCGCCCACCC	CGACGCCGCG	GCCGCCGCTGCC	-3'	C-C
	3'-	GGGGCGGGTGGG	GCTGCGGCGC	CGGCGGCGACGG	-5'	
INPP5D-2	5'-	CCCCGCCCACCC	CGACGCC	GCGGCCGCCGCT	-3'	C-C
	3'-	GGGGCGGGTGGG	GCTGCGG	CGCCGGCGGCGA	-5'	
DVL3	5'-	ACCACCAGCTTC	TTTGACTCA	GATGAGGATGAC	-3'	C-C
	3'-	TGGTGGTCGAAG	AAACTGAGT	CTACTCCTACTG	-5'	
CCR5-1	5'-	GTCATCCTCATC	CTGAT	AAACTGCAAAAG	-3'	C-C
	3'-	CAGTAGGAGTAG	GACTA	TTTGACGTTTTC	-5'	
CCR5-2	5'-	GTCATCCTCATC	CTGATAAA	CTGCAAAAGGCT	-3'	C-C
	3'-	CAGTAGGAGTAG	GACTATTT	GACGTTTTCCGA	-5'	
TRAC-CC	5'-	GTGATTGGGTTC	CGAATCCTCCT	CCTGAAAGTGGCCGG	-3'	C-C
	3'-	CACTAACCCAAG	GCTTAGGAGGA	GGACTTTCACCGGCC	-5'	
TRAC-NC	5'-	TGTCAGTGATTGGGTT	CCGAATCCTCCT	CCTGAAAGTGGCCGG	-3'	N-C
	3'-	ACAGTCACTAACCCAA	GGCTTAGGAGGA	GGACTTTCACCGGCC	-5'	

Supplementary table 5. Amino acid sequences of mitoZFDs.

• C type: MTS–FLAG tag–NES–Zinc finger protein–24aa linker–DddA$_{tox}$ half–4aa linker–UGI

• N type: MTS–HA tag–NES–DddA$_{tox}$ half–24aa linker–Zinc finger protein–4aa linker–UGI

- MTS (Mitochondrial Targeting Sequence of human mitochondrial ATP synthase F1β subunit)<br>MLGFVGRVAAAPASGALRRLTPSASLPPAQLLLRAAPTAVHPVRDYAAQ
- FLAG tag (C type)<br>DYKDDDDK<br><br>- HA tag (N type)<br>YPYDVPDYA
- NES (Nuclear export signal)<br>VDEMTKKFGTLTIHDTEK (Minute virus of mice; MVM NES)
- Split-DddA$_{tox}$ G1397-N<br>GSYALGPYQISAPQLPAYNGQTVGTFYYVNDAGGLESKVFSSGGPTPYPNYANAG HVEGQSALFMRDN GISEGLVFHNNPEGTCGFCVNMTETLLPENAKMTVVPPEG<br><br>- Split-DddA$_{tox}$ G1397-C<br>AIPVKRGATGETKVFTGNSNSPKSPTKGGC
- 4aa linker<br>SGGS
- UGI<br>TNLSDIIEKETGKQLVIQESILMLPEEVEEVIGNKPESDILVHTAYDESTDENVMLLT SDAPEYKPWA LVIQDSNGENKIKML

- ZFP

[De novo designed using the Toolgen set of zinc finger modules]
 * Zinc fingers were linked by TGEKP linker (ZF1-linker-ZF2-linker-ZF3-linker-ZF4).
 * ND2-targeting mitoZFDs with QQ variants were generated by replacing R or K, shown in red, with Q.

	Type	F1	F2	F3	F4
ND1_ Left	C	FQCRICMRNFSDS GNLRVHIRTH	YKCPDCGKSFSQS SSLIRHQRTH	YECDHCGKSFSQS SHLNVHKRTH	YRCKYCDRSFSISS NLQRHVRNIH
ND1_ Right	N	YKCPECGKSFSTK NSLTEHQRTH	YKCPECGKSFSSK KALTEHQRTH	YECNYCGKTFSVS STLIRHQRIH	YRCKYCDRSFSISS NLQRHVRNIH
ND2_ Left	C	YKCPECGKSFSRE DNLHTHQRTH	YHCDWDGCGWKF ARSDELTRHYRKH	YKCPECGKSFSRE DNLHTHQRTH	YRCKYCDRSFSISS NLQRHVRNIH
ND2_ Right	N	YECDHCGKSFSQS SHLNVHKRTH	YHCDWDGCGWKF ARSDELTRHYRKH	YKCPDCGKSFSQS SSLIRHQRTH	YKCGQCGKFYSQV SHLTRHQKIH
Cox1_Left	C	YECHDCGKSFRQS THLTQHRRIH	YSCGICGKSFSDS SAKRRHCILH	YECHDCGKSFRQS THLTQHRRIH	YKCPDCGKSFSQS SSLIRHQRTH
Cox1_Right	N	YKCPDCGKSFSQS SSLIRHQRTH	YKCGQCGKFYSQV SHLTRHQKIH	YKCPDCGKSFSQS SSLIRHQRTH	YECDHCGKSFSQS SHLNVHKRTH
Cox2_Left	C	YKCPECGKSFSDP GHLVRHQRTH	YRCKYCDRSFSISS NLQRHVRNIH	YRCKYCDRSFSISS NLQRHVRNIH	YKCPECGKSFSRE DNLHTHQRTH

Cox2_Right	N	YGCHLCGKAFSKS SNLRRHEMIH	FQCKTCQRKFSRS DHLKTHTRTH	YKCGQCGKFYSQV SHLTRHQKIH	YKCPECGKSFSRE DNLHTHQRTH
ND4L_Left	C	YKCPECGKSFSDP GHLVRHQRTH	YKCPECGKSFSRE DNLHTHQRTH	YKCPECGKSFSRE DNLHTHQRTH	YRCKYCDRSFSDS SNLQRHVRNIH
ND4L_Right	C	YKCPECGKSFSDP GHLVRHQRTH	FHCGYCEKSFSVK DYLTKHIRTH	YKCPECGKSFSSP ADLTRHQRTH	FQCRICMRNFSDS GNLRVHIRTH
ND4_ Left	C	FHCGYCEKSFSVK DYLTKHIRTH	YECVQCGKGFTQS SNLITHQRVH	YTCSYCGKSFTQS NTLKQHTRIH	YRCKYCDRSFSISS NLQRHVRNIH
ND4_ Right	C	FHCGYCEKSFSVK DYLTKHIRTH	YKCPECGKSFSTH LDLIRHQRTH	YKCPECGKSFSSK KALTEHQRTH	FQCRICMRNFSDS GNLRVHIRTH
ND5_ Left	C	FHCGYCEKSFSVK DYLTKHIRTH	YECDHCGKSFSQS SHLNVHKRTH	FQCRICMRNFSDS GNLRVHIRTH	YECDHCGKSFSQS SHLNVHKRTH
ND5_ Right	N	FACPECPKRFMRS DNLTQHIKTH	YECNYCGKTFSVS STLIRHQRIH	YECDHCGKAFSVS SNLNVHRRIH	YECDHCGKAFSVS SNLNVHRRIH
ND5-2_Left	C	YECHDCGKSFRQS THLTQHRRIH	YECHDCGKSFRQS THLTQHRRIH	YKCGQCGKFYSQV SHLTRHQKIH	YHCDWDGCGWKF ARSDELTRHYRKH
ND5-2_Right	N	YRCEECGKAFRWP SNLTRHKRIH	YKCPECGKSFSRE DNLHTHQRTH	YGCHLCGKAFSKS SNLRRHEMIH	YKCPECGKSFSRE DNLHTHQRTH
CYB_ Left	C	FECKDCGKAFIQKS NLIRHQRTH	FHCGYCEKSFSVK DYLTKHIRTH	YTCSDCGKAFRDK SCLNRHRRTH	YTCSYCGKSFTQS NTLKQHTRIH
CYB_Right	C	YTCSYCGKSFTQS NTLKQHTRIH	YKCDECGKNFTQS SNLIVHKRIH	YKCPECGKSFSDP GHLVRHQRTH	YECDHCGKSFSQS SHLNVHKRTH

Supplementary table 6. DNA sequences of zinc finger binding sites and the spacer region of mitoZFDs.
The ZFD binding sites are indicated in green.

		Left-ZFD binding region	Spacer region	Right-ZFD binding region		ZFD configuration
ND1	5'-	GTTTACTCAATC	CTCTGATC	AGGGTGAGCATC	-3'	C-N
	3'-	CAAATGAGTTAG	GAGACTAG	TCCCACTCGTAG	-5'	
ND2	5'-	CTACGCCTAATC	TACTCCACCTCAA	TCACACTACTCC	-3'	C-N
	3'-	GATGCGGATTAG	ATGAGGTGGAGTT	AGTGTGATGAGG	-5'	
Cox1	5'-	TCTGACTCTTAC	CTCCCTCTCTCC	TACTCCTGCTCG	-3'	C-N
	3'-	AGACTGAGAATG	GAGGGAGAGAGG	ATGAGGACGAGC	-5'	
Cox2	5'-	GCCATCATCCTA	GTCCTCATCGCC	CTCCCATCCCTA	-3'	C-N
	3'-	CGGTAGTAGGAT	CAGGAGTAGCGG	GAGGGTAGGGAT	-5'	
ND4L	5'-	GCCCTACTAGTC	TCAATCTCC	AACACATATGGC	-3'	C-C
	3'-	CGGGATGATCAG	AGTTAGAGG	TTGTGTATACCG	-5'	
ND4	5'-	ATATTTTATATC	TTCTTCGA	AACCACACTTAT	-3'	C-C
	3'-	TATAAAATATAG	AAGAAGCT	TTGGTGTGAATA	-5'	
ND5	5'-	ATATCGGTTTCA	TCCTCGC	CTTAGCATGATT	-3'	C-N
	3'-	TATAGCCAAAGT	AGGAGCG	GAATCGTACTAA	-5'	
ND5-2	5'-	TCTTCTTCCCAC	TCATCCTA	ACCCTACTCCTA	-3'	C-N
	3'-	AGAAGAAGGGTG	AGTAGGAT	TGGGATGAGGAT	-5'	
CYB	5'-	TTCATAGGCTAT	GTCCTCCCG	TGAGGCCAAATA	-3'	C-C
	3'-	AAGTATCCGATA	CAGGAGGGC	ACTCCGGTTTAT	-5'	

Supplementary table 7. ZFD-GST sequences for protein purification.

• C type: SV40 NLS [or 4x SV40 NLS]–Zinc finger protein–24aa linker–$DddA_{tox}$ half–4aa linker–UGI–9aa linker– thrombin site–GST

• N type: SV40 NLS [or 4x SV40 NLS]–$DddA_{tox}$ half–24aa linker–Zinc finger protein–4aa linker–UGI–9aa linker– thrombin site–GST

- SV40 NLS<br>PKKKRKV<br><br>- 4xSV40 NLS with GGS linker<br>PKKKRKV GGS PKKKRKV GGS PKKKRKV GGS PKKKRKV
- 24aa linker<br>SGTPHEVGVYTLSGTPHEVGVYTL
- Split-$DddA_{tox}$ G1397-N<br>GSYALGPYQISAPQLPAYNGQTVGTFYYVNDAGGLESKVFSSGGPTPYPNYANAG<br>HVEGQSALFMRDNGISEGLVFHNNPEGTCGFCVNMTETLLPENAKMTVVPPEG<br><br>- Split-$DddA_{tox}$ G1397-C<br>AIPVKRGATGETKVFTGNSNSPKSPTKGGC
- 4aa linker<br>SGGS
- UGI<br>TNLSDIIEKETGKQLVIQESILMLPEEVEEVIGNKPESDILVHTAYDESTDENVMLLT<br>SDAPEYKPWA LVIQDSNGENKIKML
- 9aa linker<br>GGSGGGSGG
- Thrombin site<br>LVPRGS
- GST<br>MSPILGYWKIKGLVQPTRLLLEYLEEKYEEHLYERDEGDKWRNKKFELGLEFPNL<br>PYYIDGDVKLTQSMAIIRYIADKHNMLGGCPKERAEISMLEGAVLDIRYGVSRIAY<br>SKDFETLKVDFLSKLPEMLKMFEDRLCHKTYLNGDHVTHPDFMLYDALDVVLYM<br>DPMCLDAFPKLVCFKKRIEAIPQIDKYLKSSKYIAWPLQGWQATFGGGDHPPK
- ZFP<br><br>TRAC-Left (N type) [Adapted from Paschon, D.E. et al., 2019]<br>FQCRICMRKFATSGSLTRHTKIHTGEKPFQCRICMRNFSRSDHLSTHIRTHTGEKPF<br>ACDICGRKFATSSNRTKHTKIHTHPRAPIPKPFQCRICMRNFSRSDNLSEHIRTHTGE<br>KPFACDICGRKFAWHSSLRVHTKIHLR<br><br>TRAC-Right (C type) [From Paschon, D.E. et al., 2019]<br>GIHGVPAAMAERPFQCRICMRNFSRSDHLSTHIRTHTGEKPFACDICGRKFADRSH<br>LARHTKIHTGSQKPFQCRICMRKFALKQHLNEHTKIHTGEKPFQCRICMRNFSQSG<br>NLARHIRTHTGEKPFACDICGRKFAHNSSLKDHTKIHLR

Supplementary table 8. The DNA sequence of the PCR amplicon for the *in vitro* activity test of the ZFD protein.

The ZFD binding sites are indicated in green. The spacer is indicated in red.

ACATCACCTCCCACAACGAGGACTACACCATCGTGGAACAGTACGAGCGCGCCGAGG<br>GCCGCCACTCCACCGGCGCCGAATTACCTAAACTTTCAAAACC**TGTCAGTGATTGGG**<br>**TTCCGAATCCTCCTCCTGAAAGTGGCCGG**GTTTAATCTGCTCATGACGCTGCGGATC<br>GCTGAGGCAGTGAGCAAGGGCGAGGAGCTGTTCACCGGGGTGGTGCCCATCCTGGTC<br>GAGCTGGACGGCGACGTAAACGGCCACAAGTTCAGCGTGTCCGGCGAGGGCGAGGGC<br>GATGCCACCTACGGCAAGCTGACCCTGAAGTTCATCTGCACCACCGGCAAGCTGCCCG<br>TGCCCTGGCCCACCCTCGTGACCACCCTGACCTACGGCGTGCAGTGCTTCAGCCGCTAC<br>CCCGACCACATGAAGCAGCACGACTTCTTCAAGTCCGCCATGCCCGAAGGCTACGTCC<br>AGGAGCGCACCATCTTCTTCAAGGACGACGGCAACTACAAGACCCGCGCCGAGGTGA<br>AGTTCGAGGGCGACACCCTGGTGAACCGCATCGAGCTGAAGGGCATCGACTTCAAGG<br>AGGACGGCAACATCCTGGGGCACAAGCTGGAGTACAACTACAACAGCCACAACGTCT<br>ATATCATGGCCGACAAGCAGAA

Supplementary table 9. Amino acid sequence of COX2 targeting DdCBE.

• Left-SOD2 MTS-3×HA-N terminal domain-TALE repeat-C terminal half domain- $DddA_{tox}$ half-UGI

• Right-COX8A MTS-3×FLAG-N terminal domain- TALE repeat-C terminal half domain- $DddA_{tox}$ half-UGI

COX2 Left TALE<br>MALSRAVCGTSRQLAPVLGYLGSRQKHSLPDYPYDVPDYAGYPYDVPDYAGYPY<br>DVPDYAGIRIQDLRTLGYSQQQQEKIKPKVRSTVAQHHEALVGHGFTHAHIVALS<br>QHPAALGTVAVKYQDMIAALPEATHEAIVGVGKQWSGARALEALLTVAGELRGP<br>PLQLDTGQLLKIAKRGGVTAVEAVHAWRNALTGAPL<br>LNLTPDQVVAIAS**NN**GGKQALETVQRLLPVLCQAHGLTPDQVVAIAS**HD**GGKQA<br>LETVQRLLPVLCQDHGLTPDQVVAIAS**HD**GGKQALETVQRLLPVLCQAHGLTPAQ<br>VVAIAS**HD**GGKQALETVQRLLPVLCQAHGLTPDQVVAIAS**NN**GGKQALETVQRL<br>LPVLCQDHGLTPDQVVAIAS**HD**GGKQALETVQRLLPVLCQDHGLTPAQVVAIAS**H**<br>**D**GGKQALETVQRLLPVLCQDHGLTPDQVVAIAS**NI**GGKQALETVQRLLPVLCQDH<br>GLTPAQVVAIAS**NG**GGKQALETVQRLLPVLCQAHGLTPAQVVAIAS**HD**GGKQAL<br>ETVQRLLPVLCQDHGLTPDQVVAIAS**NI**GGKQALETVQRLLPVLCQDHGLTPAQV<br>VAIAS**NG**GGKQALETVQRLLPVLCQAHGLTPDQVVAIAS**HD**GGKQALETVQRLLP<br>VLCQAHGLTPAQVVAIAS**HD**GGKQALETVQRLLPVLCQDHGLTPAQVVAIAS**NG**<br>GGKQALETVQRLLPVLCQDHGLTPDQVVAIAS**NI**GGKQALETVQRLLPVLCQDHG<br>LTPAQVVAIAS**NN**GGKQALETVQRLLPVLCQDH<br>GLTPEQVVAIASNGGGKQALESIVAQLSRPDPALAALTNDHLVALACLGGRPALD<br>AVKKGLGGS<br>(RVDs are shown in bold letters)
COX2 Right TALE<br>MASVLTPLLLRGLTGSARRLPVPRAKIHSLDYKDHDGDYKDHDIDYKDDDDKGIR<br>IQDLRTLGYSQQQQEKIKPKVRSTVAQHHEALVGHGFTHAHIVALSQHPAALGTV<br>AVKYQDMIAALPEATHEAIVGVGKQWSGARALEALLTVAGELRGPPLQLDTGQL<br>LKIAKRGGVTAVEAVHAWRNALTGAPL<br>NLTPDQVVAIAS**NN**GGKQALETVQRLLPVLCQAHGLTPAQVVAIAS**NG**GGKQAL<br>ETVQRLLPVLCQDHGLTPAQVVAIAS**NI**GGKQALETVQRLLPVLCQAHGLTPAQV<br>VAIAS**NI**GGKQALETVQRLLPVLCQDHGLTPDQVVAIAS**NI**GGKQALETVQRLLPV<br>LCQDHGLTPDQVVAIAS**NN**GGKQALETVQRLLPVLCQAHGLTPAQVVAIAS**NN**G<br>GKQALETVQRLLPVLCQDHGLTPAQVVAIAS**NI**GGKQALETVQRLLPVLCQAHGL<br>TPEQVVAIAS**NG**GGKQALETVQRLLPVLCQAHGLTPAQVVAIAS**NN**GGKQALETV<br>QRLLPVLCQAHGLTPDQVVAIAS**HD**GGKQALETVQRLLPVLCQDHGLTPAQVVAI<br>AS**NN**GGKQALETVQRLLPVLCQAHGLTPAQVVAIAS**NG**GGKQALETVQRLLPVL<br>CQDHGLTPAQVVAIAS**NI**GGKQALETVQRLLPVLCQAHGLTPDQVVAIAS**NN**GGK<br>QALETVQRLLPVLCQAHGLTPAQVVAIAS**NN**GGKQALETVQRLLPVLCQAHGLTP<br>DQVVAIAS**NN**GGKQALETVQRLLPVLCQDHGLTPAQVVAIAS**NI**GGKQALETVQR<br>LLPVLCQAH<br>GLTPEQVVAIASNGGGKQALESIVAQLSRPDPALAALTNDHLVALACLGGRPALD<br>AVKKGLGGS<br>(RVDs are shown in bold letters)
-Split-$DddA_{tox}$ G1397-N

GSYALGPYQISAPQLPAYNGQTVGTFYYVNDAGGLESKVFSSGGPTPYPNYANAG HVEGQSALFMRDNGISEGLVFHNNPEGTCGFCVNMTETLLPENAKMTVVPPEG<br><br>-Split-DddA$_{tox}$ G1397-C<br>AIPVKRGATGETKVFTGNSNSPKSPTKGGC
-UGI<br>TNLSDIIEKETGKQLVIQESILMLPEEVEEVIGNKPESDILVHTAYDESTDENVMLLT SDAPEYKPWA LVIQDSNGENKIKML

Supplementary table 10. List of primers used for targeted deep sequencing.

Target Site	1st-F	1st-R	2nd-F	2nd-R
pTarget	GACCGACATCAAGCTGGACA	GCATGGCGGACTTGAAGAAG	ACACTCTTTCCCTACACGACGCTCTTCCGATCTACATCACCTCCCACAACGAG	GTGACTGGAGTTCAGACGTGTGCTCTTCCGATCTCACCCCGGTGAACAGCTC
CCR5	TTATGCACAGGGTGGAACAA	AAACACAGCATGGACGACAG	ACACTCTTTCCCTACACGACGCTCTTCCGATCTTATACATCGGAGCCCTGCCAA	GTGACTGGAGTTCAGACGTGTGCTCTTCCGATCTAGCATAGTGAGCCCAGAAGG
TRAC	ACAGAAGCTGCAAGGGACAG	AACTGAGGCGGCTGAAATGA	ACACTCTTTCCCTACACGACGCTCTTCCGATCTTGGGACATGCAAGCCCATAA	GTGACTGGAGTTCAGACGTGTGCTCTTCCGATCTCAGAGCTTAGGATGCACCCA
MFAP1	AGGCCACAGGAACAAGATCG	ACCGACTCGACTTCCTAGCT	ACACTCTTTCCCTACACGACGCTCTTCCGATCTGAGAGGTCTGGAGGTTGCTG	GTGACTGGAGTTCAGACGTGTGCTCTTCCGATCTCTTCGTTGACGTTGCTGGTG
CCDC28B	TTTGACCTCTTCACGCCTCC	GAACGAAGCCCCTGTCTGAA	ACACTCTTTCCCTACACGACGCTCTTCCGATCTAAAAGATCGGGAGGTGCCTG	GTGACTGGAGTTCAGACGTGTGCTCTTCCGATCTCCGGCCAGAGTGGAAATCAT
INPP5D	TCCCAGGAAGGACCAGGAAT	CACATACGCACTCACGCATG	ACACTCTTTCCCTACACGACGCTCTTCCGATCTGGGACAAGAGCCAAGGGAAG	GTGACTGGAGTTCAGACGTGTGCTCTTCCGATCTCTCGGTGTTGTCGCGGTAG
KDM4B	CTGTGGCTCTTCTCACAGGG	AGCCGAGCATCTCTAACAGC	ACACTCTTTCCCTACACGACGCTCTTCCGATCTTCAGGCACCGTCCACATTTT	GTGACTGGAGTTCAGACGTGTGCTCTTCCGATCTCAGGAAGTGGGGGAAGGAAG
Numbl	TTCTGACCTCAAGCGATCCG	AACCCAGTACCTAGCGGCTA	ACACTCTTTCCCTACACGACGCTCTTCCGATCTTTGAGTCTCTGCACCTCTGC	GTGACTGGAGTTCAGACGTGTGCTCTTCCGATCTGACGGGAGAAGGAATGTGGG
DVL3	CCCAGCTACCCACCTTCTTG	CTGTTCTGTGGAGCTGCTGA	ACACTCTTTCCCTACACGACGCTCTTCCGATCTAGCAACCCGGCTAAATGGAA	GTGACTGGAGTTCAGACGTGTGCTCTTCCGATCTACAAAGCCCATAAGCCTCCC
ND1	GGTTCGGTTGGTCTCTGCTA	ATGGCCAACCTCCTACTCCT	ACACTCTTTCCCTACACGACGCTCTTCCGATCTAAGGGTGGAGAGGTTAAAGGAG	GTGACTGGAGTTCAGACGTGTGCTCTTCCGATCTCCCTGGTCAACCTCAACCTA
ND2	GCCAGTTGATTAGGGTGCTT	ATTCCATCCACCCTCCTCTC	ACACTCTTTCCCTACACGACGCTCTTCCGATCTGTGGTAAGGGCGATGAGTGT	GTGACTGGAGTTCAGACGTGTGCTCTTCCGATCTGGCCATTATCGAAGAATTCAC
Cox1	TGAAATTGATGGCCCCTAAG	TACCTCACCCCCACTGATGT	ACACTCTTTCCCTACACGACGCTCTTCCGATCTGCTCCAGGGTGGGAGTAGTT	GTGACTGGAGTTCAGACGTGTGCTCTTCCGATCTCACAGCCCATGCATTTGTAA
Cox2	ATGGGCATGAAACTGTGGTT	TTCATGATCACGCCCTCATA	ACACTCTTTCCCTACACGACGCTCTTCCGATCTCATTGGTGGCCAATTGATTT	GTGACTGGAGTTCAGACGTGTGCTCTTCCGATCTGTCCTGTATGCCCTTTTCCT
ND4L	CCAAATGCCCCTCATTTACA	TGTGAGGGGTAGGAGTCAGG	ACACTCTTTCCCTACACGACGCTCTTCCGATCTGCCAATATTGTGCCTATTGC	GTGACTGGAGTTCAGACGTGTGCTCTTCCGATCTTGTGGGTGGTTGTGTTGATT
ND4	AGTTTTAAGAGTACTGCGGCAAG	CCTACCCCTCACAATCATGG	ACACTCTTTCCCTACACGACGCTCTTCCGATCTGAGTAGGGGAAGGGAGCCTA	GTGACTGGAGTTCAGACGTGTGCTCTTCCGATCTCAACGCCACTTATCCAGTGA
ND5	TGGAGACCTAATTGGGCTGA	CATTGTCGCATCCACCTTTA	ACACTCTTTCCCTACACGACGCTCTTCCGATCTTGAGGCTTGGATTAGCGTTTA	GTGACTGGAGTTCAGACGTGTGCTCTTCCGATCTCAACTGTTCATCGGCTGAGAG
ND5-2	AGGTAGGATTGGTGCTGTGG	CCCTCGCTGTCACTTTCCTA	ACACTCTTTCCCTACACGACGCTCTTCCGATCTGAGAGGGGTCAGGGTTGATT	GTGACTGGAGTTCAGACGTGTGCTCTTCCGATCTCACCAAATCTCCACCTCCAT
CYB	AGGGGTTGGCTAGGGTATAA	CCCACATCACTCGAGACGTA	ACACTCTTTCCCTACACGACGCTCTTCCGATCTTGCAATAATGAAGGGCAAGA	GTGACTGGAGTTCAGACGTGTGCTCTTCCGATCTTCTACTCAGAAACCTGAAACATCG
pseudo-ND2-target (chr1)	ACCTTCAGCAAGGTCAAAGG	CAGTGTGGTAAGGGCGATG	ACACTCTTTCCCTACACGACGCTCTTCCGATCTGTGGTAAGGGCGATGAGTGT	GTGACTGGAGTTCAGACGTGTGCTCTTCCGATCTGGCCATTATCGAAGAATTCAC
pseudo-ND4L-target-1 (chr5)	GTTAAGTTACAGGTTAAACCCCG	GGTTGATGAAGGTGGCAATGATC	ACACTCTTTCCCTACACGACGCTCTTCCGATCTCTCCCTCTTAGCCAACATCG	GTGACTGGAGTTCAGACGTGTGCTCTTCCGATCTAATTAGGCTGTGGGTGGTTG

pseudo-ND4L-target-2 (chr5)	TTCACCACGTTAGCCAGGAT	GTGGTTCATTGGATAAGTGGC	ACACTCTTTCCCTACACGACGCTCTTCCGATCTCCCACTCCCTCTTAGCCAAT	GTGACTGGAGTTCAGACGTGTGCTCTTCCGATCTGGCTGTGAGTGGTTGTGTTG
pseudo-ND4L-target-3 (chrX)	AGGCTAAGCGTTTTGAGCTG	CTGGGCAATAGAGCAAGACC	ACACTCTTTCCCTACACGACGCTCTTCCGATCTCCCACTCCCTCTTAGCCAAT	GTGACTGGAGTTCAGACGTGTGCTCTTCCGATCTGGCTGTGAGTGGTTGTGTTG
