# Supplementary material for: Nuclear and mitochondrial DNA editing in human cells with zinc finger deaminases
Source: Nat Commun. 2022 Jan 18;13:366. doi: 10.1038/s41467-022-27962-0 (PMC8766470; doi:10.1038/s41467-022-27962-0)
Supplement: Supplementary file 2 — Reporting Summary [file 41467_2022_27962_MOESM2_ESM.pdf]

nature portfolio

Corresponding author(s): Jin-Soo Kim
NCOMMS-21-34580-T

Last updated by author(s): Dec 17, 2021

Reporting Summary

Nature Portfolio wishes to improve the reproducibility of the work that we publish. This form provides structure for consistency and transparency in reporting. For further information on Nature Portfolio policies, see our Editorial Policies and the Editorial Policy Checklist.

Statistics

For all statistical analyses, confirm that the following items are present in the figure legend, table legend, main text, or Methods section.

n/a	Confirmed	
☐	☒	The exact sample size (n) for each experimental group/condition, given as a discrete number and unit of measurement
☐	☒	A statement on whether measurements were taken from distinct samples or whether the same sample was measured repeatedly
☐	☒	The statistical test(s) used AND whether they are one- or two-sided<br>*Only common tests should be described solely by name; describe more complex techniques in the Methods section.*
☒	☐	A description of all covariates tested
☒	☐	A description of any assumptions or corrections, such as tests of normality and adjustment for multiple comparisons
☐	☒	A full description of the statistical parameters including central tendency (e.g. means) or other basic estimates (e.g. regression coefficient) AND variation (e.g. standard deviation) or associated estimates of uncertainty (e.g. confidence intervals)
☐	☒	For null hypothesis testing, the test statistic (e.g. F, t, r) with confidence intervals, effect sizes, degrees of freedom and P value noted<br>*Give P values as exact values whenever suitable.*
☒	☐	For Bayesian analysis, information on the choice of priors and Markov chain Monte Carlo settings
☒	☐	For hierarchical and complex designs, identification of the appropriate level for tests and full reporting of outcomes
☒	☐	Estimates of effect sizes (e.g. Cohen's d, Pearson's r), indicating how they were calculated

Our web collection on statistics for biologists contains articles on many of the points above.

Software and code

Policy information about availability of computer code

Data collection	Targeted deep sequencing and whole-mitochondrial DNA sequencing were performed using MiniSeq system (Illumina).
Data analysis	Base editing frequencies and indel frequencies from targeted deep sequencing data were calculated with source code (https://github.com/ibs-cge/maund). For analysis of whole-mtDNA sequencing, a description is provided in [Analysis of mitochondrial genome-wide DNA editing] part of the Methods.<br>GraphPad Prism 8, BWA (v.0.7.17), SAMtools (v.1.9), REDItools (v.1.2.1), Microsoft Excel 2016, PowerPoint 2016, and Adobe Illustrator CS6 were used for data analysis and visualization.

For manuscripts utilizing custom algorithms or software that are central to the research but not yet described in published literature, software must be made available to editors and reviewers. We strongly encourage code deposition in a community repository (e.g. GitHub). See the Nature Portfolio guidelines for submitting code & software for further information.

Data

Policy information about availability of data

All manuscripts must include a data availability statement. This statement should provide the following information, where applicable:

- Accession codes, unique identifiers, or web links for publicly available datasets
- A description of any restrictions on data availability
- For clinical datasets or third party data, please ensure that the statement adheres to our policy

DNA sequencing data will be deposited in the National Center for Biotechnology Information(NCBI) Sequence Read Archive (SRA) database with BioProject accession code PRJNA756903.
(Reviewer link; https://dataview.ncbi.nlm.nih.gov/object/PRJNA756903?reviewer=ro0jmvflr8dc06u7vl5qvsv4on)

Field-specific reporting

Please select the one below that is the best fit for your research. If you are not sure, read the appropriate sections before making your selection.

- [x] Life sciences
- [ ] Behavioural & social sciences
- [ ] Ecological, evolutionary & environmental sciences

For a reference copy of the document with all sections, see nature.com/documents/nr-reporting-summary-flat.pdf

Life sciences study design

All studies must disclose on these points even when the disclosure is negative.

Sample size	Sample sizes were determined based on previous publications of our and other groups generating reproducible results for genome editing experiments.
Data exclusions	No data were excluded from the analyses.
Replication	All experiments were performed from individual biological replicates (n = 2 or 3).<br>Only one sample in the pTarget library experiment was performed in a single replicate, and all other attempts at replication were successful.
Randomization	Randomization was not relevant for this study as cultured cells were grown and analyzed under identical conditions.
Blinding	Blinding was not relevant for this study as cultured cells were grown and analyzed under identical conditions.

Reporting for specific materials, systems and methods

We require information from authors about some types of materials, experimental systems and methods used in many studies. Here, indicate whether each material, system or method listed is relevant to your study. If you are not sure if a list item applies to your research, read the appropriate section before selecting a response.

Materials & experimental systems

n/a	Involved in the study	
x		Antibodies
	x	Eukaryotic cell lines
x		Palaeontology and archaeology
x		Animals and other organisms
x		Human research participants
x		Clinical data
x		Dual use research of concern

Methods

n/a	Involved in the study	
x		ChIP-seq
x		Flow cytometry
x		MRI-based neuroimaging

Eukaryotic cell lines

Policy information about cell lines

Cell line source(s)	HEK293T (ATCC, CRL-11268), K562 (ATCC, CCL-243)
Authentication	None of the cell lines used were authenticated.
Mycoplasma contamination	Cell lines were not tested for mycoplasma contamination.
Commonly misidentified lines (See ICLAC register)	No commonly misidentified lines were used.
